# Pan-drug and drug-specific mechanisms of 5-FU, irinotecan (CPT-11), oxaliplatin, and cisplatin identified by comparison of transcriptomic and cytokine responses of colorectal cancer cells

**DOI:** 10.18632/oncotarget.28075

**Published:** 2021-09-28

**Authors:** Lindsey Carlsen, Christoph Schorl, Kelsey Huntington, Liz Hernandez-Borrero, Aakash Jhaveri, Shengliang Zhang, Lanlan Zhou, Wafik S. El-Deiry

**Affiliations:** ^1^Laboratory of Translational Oncology and Experimental Cancer Therapeutics, The Warren Alpert Medical School, Brown University, Providence, RI 02903, USA; ^2^The Joint Program in Cancer Biology, Brown University and the Lifespan Health System, Providence, RI 02903, USA; ^3^Department of Pathology and Laboratory Medicine, The Warren Alpert Medical School, Brown University, Providence, RI 02903, USA; ^4^Pathobiology Graduate Program, The Warren Alpert Medical School, Brown University, Providence, RI 02903, USA; ^5^Department of Molecular Biology, Cell Biology and Biochemistry, The Warren Alpert Medical School, Brown University, Providence, RI 02903, USA; ^6^Genomics Core Facility, Brown University, Providence, RI 02903, USA; ^7^Hematology-Oncology Division, Department of Medicine, Rhode Island Hospital and Brown University, Providence, RI 02903, USA; ^8^Cancer Center at Brown University, The Warren Alpert Medical School, Brown University, Providence, RI 02903, USA

**Keywords:** chemotherapy mechanism, colon cancer, 5-fluorouracil, irinotecan, oxaliplatin

## Abstract

Colorectal cancer (CRC) caused over 900,000 deaths worldwide in 2020. A majority of late-stage CRC patients are treated with 5-fluorouracil (5-FU) combined with either irinotecan (CPT-11), oxaliplatin, or both. Despite their widespread use, the mechanisms of efficacy and toxicity of these drugs remain incompletely understood. While previous work has investigated cellular responses to these agents individually, we directly compare the transcriptomic and cytokine profiles of HCT116 wild-type and p53−/− colorectal cancer cells treated with these drugs and report pan-drug, drug-specific, drug class-specific, p53-independent, and p53-dependent signatures. We observed downregulation of histone genes by 5-FU (that significantly correlates with improved survival in CRC patients) and upregulation of FOS and ATF3 by oxaliplatin (which may contribute to peripheral neuropathy). BTG2 was identified as a top gene upregulated by all four drugs, suggesting its critical role in the cellular response to chemotherapy in CRC. Soluble TRAILR2 (death receptor 5; DR5) is a decoy receptor for TRAIL, an apoptosis-inducing cytokine. TRAILR2 was down-regulated by oxaliplatin and 5-FU, was not affected by CPT-11, and was increased by cisplatin. There was an increase in IL-8 by oxaliplatin and increase in ferritin by cisplatin which may contribute to cancer cell survival. Novel drug-specific mechanisms of efficacy or toxicity identified in these signatures may be targeted with combination therapies or development of new targeted therapies. Together, the findings here contribute to our understanding of the molecular bases of efficacy and toxicity of chemotherapeutic agents often used for treatment of GI cancer such as CRC.

## INTRODUCTION

### Colorectal cancer: incidence, prognosis, treatment, and molecular subtypes

Colorectal cancer (CRC) is the second leading cause of cancer death worldwide and its incidence has been steadily rising in recent decades, with nearly 2 million new cases diagnosed around the world in 2020. The five-year survival rate of this disease is as low as 13% once it reaches distant organs [1]. CRC can be treated with surgical resection, radiation, targeted therapies, immunotherapy, and/or chemotherapy. Chemotherapy increases overall survival of patients by ~20 months and has remained a frontline therapy [2]. 5-fluorouracil (5-FU) is the main active drug used to treat CRC and it has been combined other drugs including oxaliplatin and irinotecan (CPT-11) in the clinic to improve outcomes [3]. Preclinical studies suggest a synergistic effect of these combinations and these findings translate to the clinic, with response rates rising to 40–50% or more when single-agent 5-FU treatment is combined with oxaliplatin or CPT-11 [4]. Advanced CRC in 2021 is treated according to genetic alterations including the microsatellite stable (MSS) groups with KRAS/NRAS mutations, BRAF mutations, and KRAS/NRAS/BRAF WT, or microsatellite unstable (MSI) [5]. Classification of patients based on certain genetic alterations has some predictive and prognostic value [6], however more work needs to be done as far as creating prognostic gene signatures to predict outcomes [7].

### Understanding of the cellular responses that mediate efficacy and toxicity of chemotherapeutics used for treatment of CRC is incomplete

The primary targets of the chemotherapeutics used for treatment of CRC are well-established. 5-FU inhibits thymidylate synthase (TS), which prevents production of deoxythymidine mono-phosphate that is essential for DNA replication and repair [8]. 5-FU has been combined with oxaliplatin and CPT-11 to improve outcomes in the clinic [3]. Oxaliplatin (and its analogue cisplatin that is not used to treat CRC) are platinum-based therapeutics that damage DNA via inter- and intra-strand crosslinks [9]. CPT-11 is a topoisomerase inhibitor that causes cytotoxic protein-linked DNA breaks [10]. Though these primary targets are well-established, the precise mechanisms by which these drugs contribute to efficacy and toxicity in cancer patients remain incompletely understood. For example, questions remain regarding the exact mechanisms downstream of 5-FU-mediated TS inhibition [11–13]. Despite identical primary targets, cisplatin and oxaliplatin have strikingly different efficacy and toxicity profiles including oxaliplatin-specific peripheral neuropathy that occurs in 30–50% of patients [14–16]. Moreover, it is well-recognized that these drugs have anticancer activities that extend beyond those driven by their primary target [9, 10, 13]. Unraveling these mechanisms is essential to improve and predict outcomes in CRC. Identifying and exploiting specific mechanisms of efficacy or toxicity of individual drugs could help improve or predict outcomes. Previous studies have investigated these drugs separately and RNA expression has been evaluated in patient samples after combination treatment, but these types of studies introduce a number of variables including cell line, time-point, drug dose, combination treatments, pre-existing conditions, and method of analysis which can make it difficult to decipher mechanistic differences across drugs [17, 18]. Direct comparison across drugs could elucidate novel drug-specific mechanisms, aiding the preclinical development of targeted therapies, refinement of existing compounds, and guiding informed combination therapies in the clinic. This type of analysis would also give clinicians a better understanding of the molecular basis of response of CRC tumors which have been exposed to these drugs.

### Tumor suppressor p53 is frequently mutated in CRC and plays an important yet incompletely understood role in the response to chemotherapy treatment

TP53 is the most frequently mutated gene in cancer and is mutated in ~50% of CRC patients. The encoded protein, p53, is a transcription factor that is activated by cell stressors such as DNA damage, oncogenic signaling, and hypoxia. p53 responds by activating its target genes which mediate cell fates relevant to the response to chemotherapy including apoptosis, cell cycle arrest, and DNA repair, among others [19]. p53 mutations generally occur late in CRC disease progression and result in increased lymphatic and vascular invasion, chemo-resistance, and a decline in prognosis. Reactivation of wild-type (WT) p53 function holds therapeutic potential, however little success has been made in this area [20]. It is possible that heterogeneity in the p53 response is partially responsible for this lack of progress. It is well-recognized that the p53 response varies across tissue, cell type, drug type, and drug dose [21–23], and that the most important p53 targets for tumor suppression may vary across cancer types [24]. Though this heterogeneity is recognized, it has not been exploited clinically. Further investigation of heterogeneity in the p53 response across chemotherapeutics used to for CRC would enhance our understanding of what the most important p53 targets are in treatment of CRC, personalize predictive/prognostic biomarkers for patients based on their p53 status, suggest potential combination therapies, and provide an explanation for drug-specific efficacy and/or toxicity across p53 status. Due to considerable variation across studies seeking to identify p53 targets [25], evaluation of heterogeneity in the p53 response is likely best achieved by direct comparison across drugs.

## RESULTS

### Drug-specific variability in the kinetics of the p53 response to 5-FU, CPT-11, oxaliplatin, and cisplatin in colorectal cancer cells

**Figure 1:**
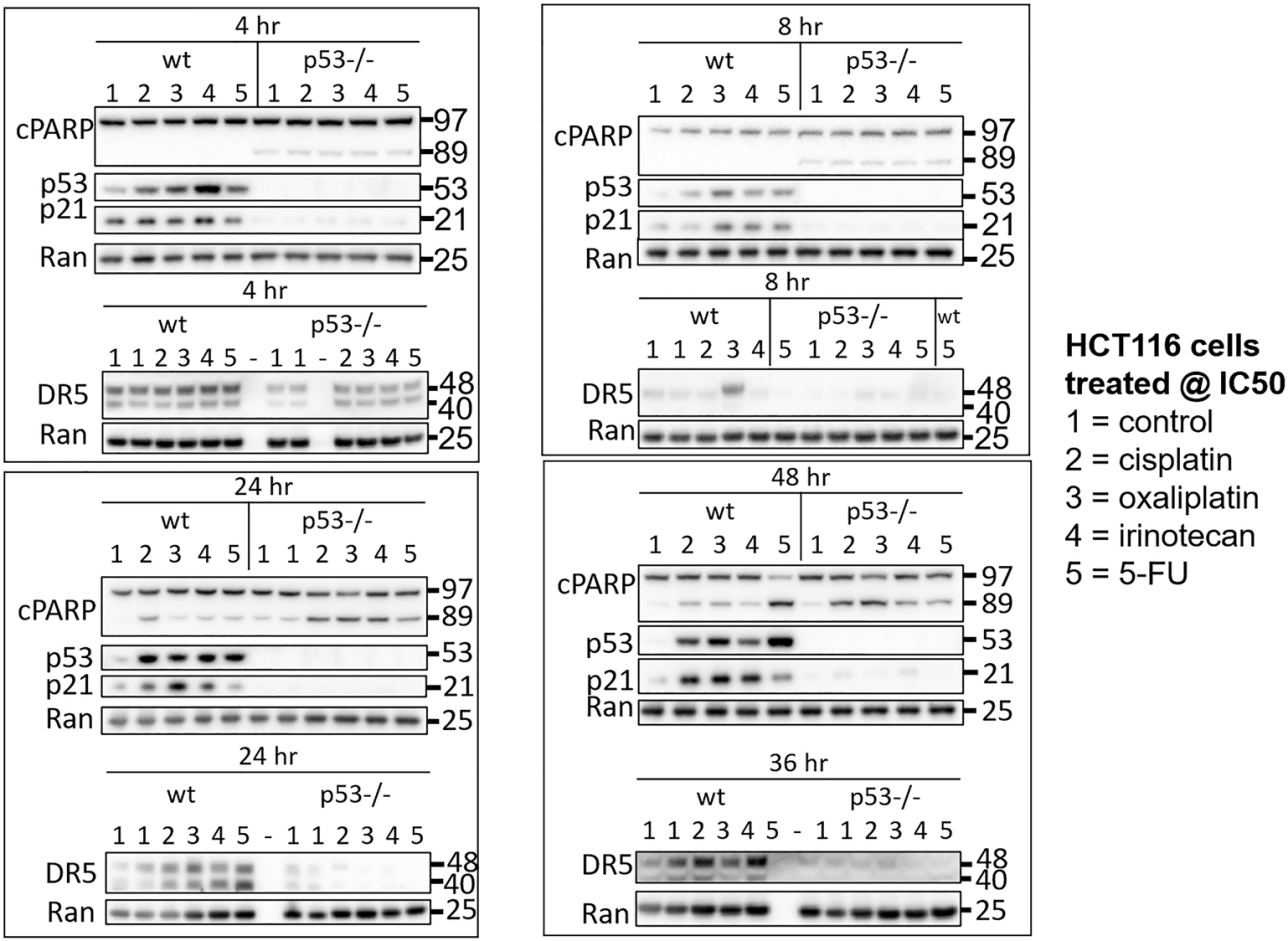
Drug-specific variability in the kinetics of the p53 response to 5-FU, CPT-11, oxaliplatin, and cisplatin in human colorectal cancer cells. HCT116 and HCT116 p53−/− cells were treated with 5-FU, CPT-11, oxaliplatin, and cisplatin at their respective IC50s at various time points.

To initially explore heterogeneity in the p53 response to drugs used for the treatment of CRC, HCT116 and HCT116 p53−/− cells were treated with 5-FU, CPT-11, and oxaliplatin at their respective IC50s. Cisplatin was also included in the analysis to investigate molecular mechanisms of varied efficacy and toxicity compared to oxaliplatin, with particular focus on oxaliplatin-induced peripheral neuropathy. Cells were harvested at multiple time points ranging from 1–48 hours and levels of p53 and two of its important downstream targets p21 and DR5 were measured in cell lysates via western blot. Variability in the kinetics of the p53 response in the wild-type cells was observed as early as 1 hour and continued out to 48 hours (Figure 1, Supplementary Figure 1A–1D). Variability in the upregulation of p53 target genes was observed across treatment conditions that induced similar amounts of PARP cleavage and similar amounts of p53, suggesting that mechanistic differences separate from drug potency and level of p53 induction play a role in regulating classical p53 targets. Similar observations were made with two other p53 targets MDM2 and GADD45A, but not BAX (Supplementary Figure 1E). These results support the idea that there is variability in the kinetics of the p53 response to different chemotherapeutic drugs. Based on the dramatic differences in regulation of these classical p53 targets at certain time points, we hypothesized that this variability would extend to much of the transcriptome.

### Pan-drug gene signature contains transcripts critical to the cellular response to chemotherapy in CRC cells and confirms importance of p53 in mediating this response

**Figure 2:**
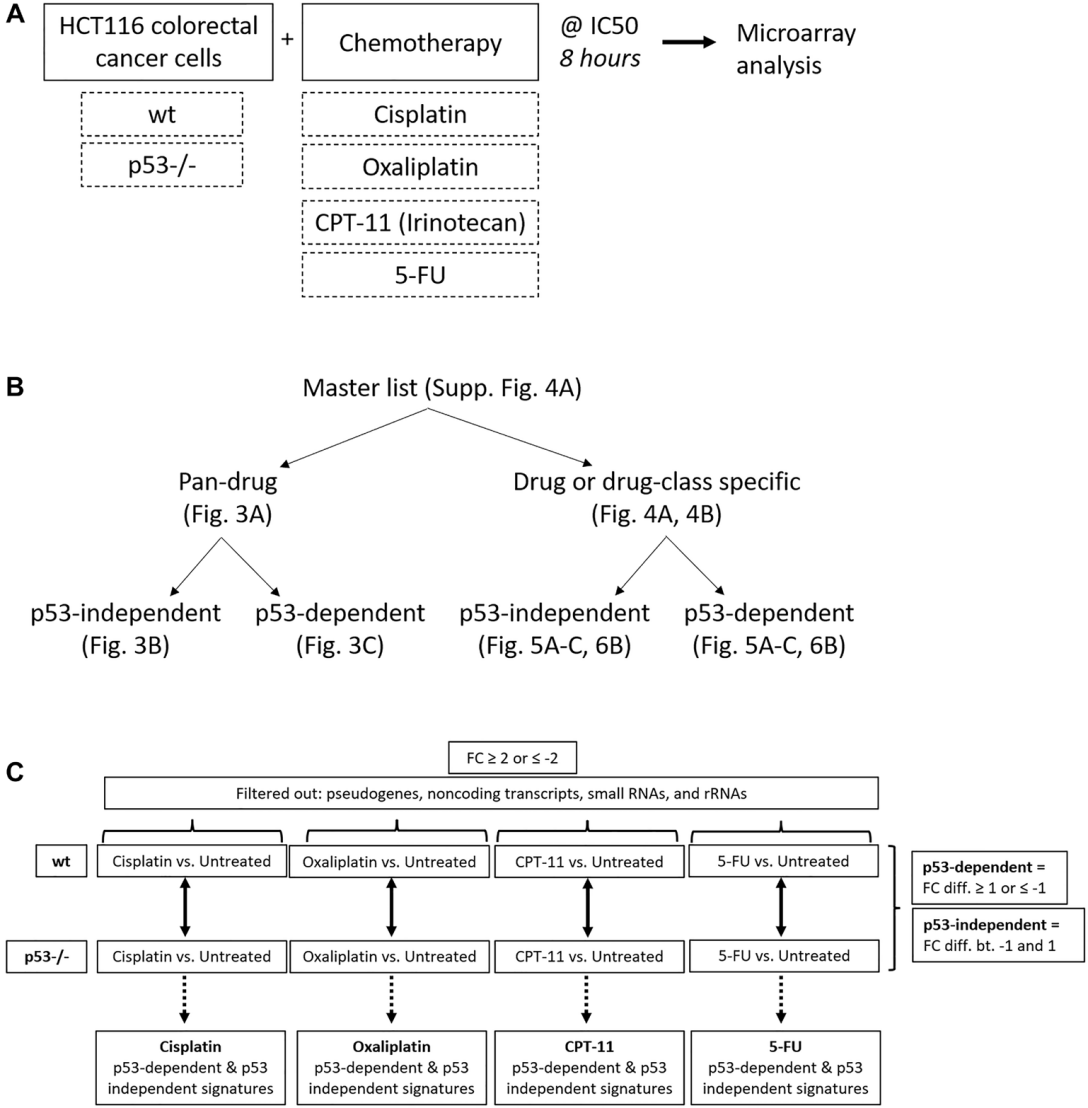
Creation of pan-drug, drug-specific, drug class-specific, p53-independent, and p53-dependent gene signatures. (**A**) Experimental design used to create gene signatures. HCT116 and HCT116 p53−/− cells were treated with cisplatin, oxaliplatin, CPT-11, or 5-FU at their IC50 for 8 hours and RNA expression relative to an untreated control was measured using microarrays. (**B**) The master gene list was divided into pan-drug, drug-specific, drug class-specific, p53-independent, and p53-dependent gene signatures. (**C**) Filtering method used to create p53-independent and -dependent gene signatures.

After identifying significant differences in upregulation of p53 and four of its target genes across drugs at the protein level, we sought to evaluate this variability on a whole-transcriptome scale using microarrays. We expected at least some portion of the p53 response to be heterogeneous across drugs, but that some elements would be critical to the response to chemotherapy and thus would be regulated across all drugs. To define a pan-drug signature and to evaluate heterogeneity in the p53 response on a whole-transcriptome scale, HCT116 and HCT116 p53−/− cells were treated with each of the four drugs at their IC50 for 8 hours, and RNA expression relative to an untreated control was measured using microarray analysis (Figure 2A). Upregulation of p53 in these samples and equal induction of cell death at this dose and time point was validated via western blot and CellTiterGlo, respectively, prior to microarray analysis (Supplementary Figure 2A, 2B). Internal control genes β-actin and GAPDH were evaluated prior to further analysis of microarray data, each of which showed no change across drug treatments and had tightly clustered triplicates (Supplementary Figure 3A). Quality control measurements were also determined to be satisfactory for further analysis (Supplementary Figure 3B).

**Figure 3:**
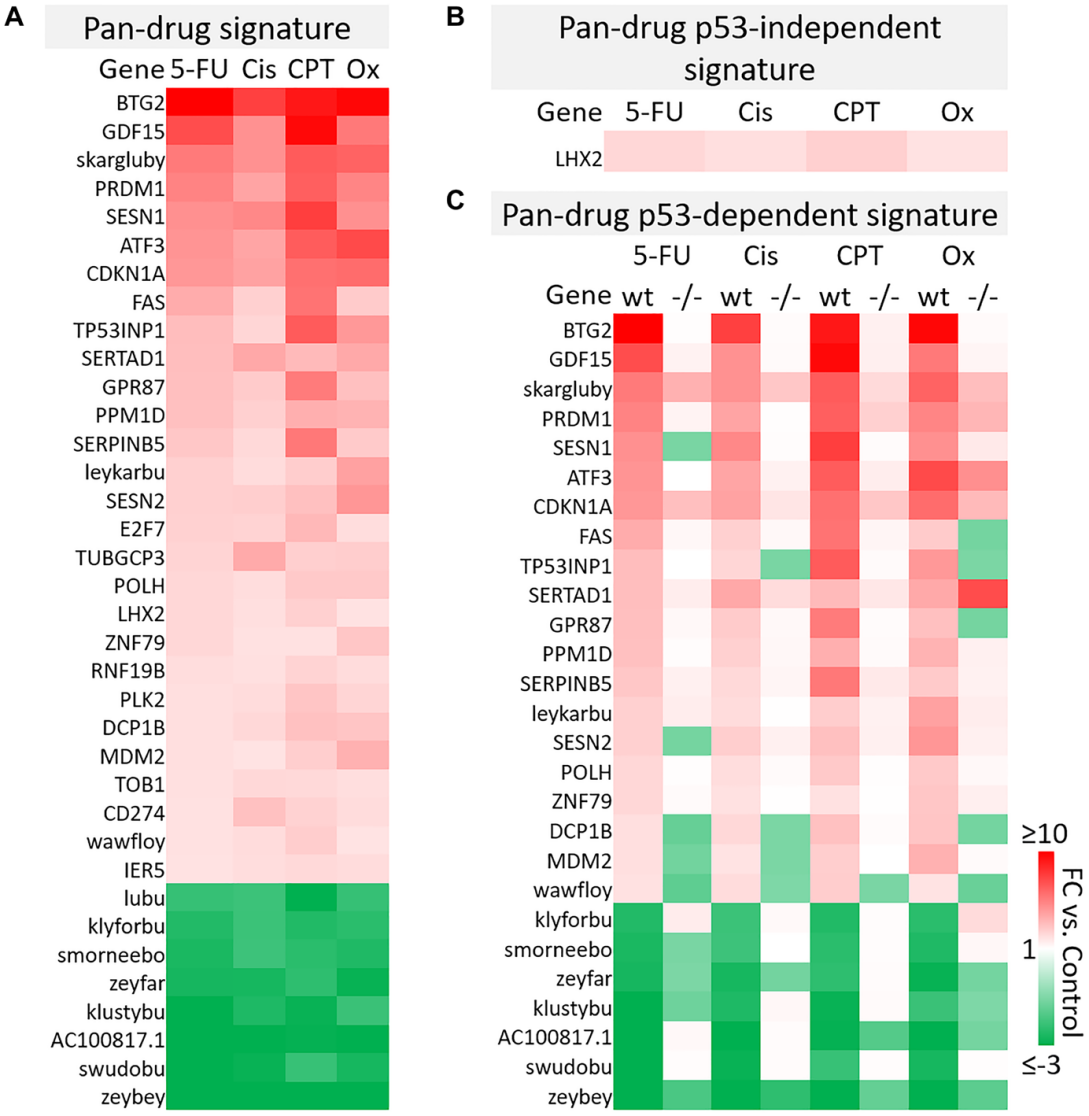
Pan-drug gene signatures after 5-FU, CPT-11, oxaliplatin, and cisplatin treatment of human colorectal cancer cells. (**A**) Pan-drug signature. 36 transcripts were up- or down-regulated (≥2 or ≤−2-fold change compared to the control, respectively) by all drug treatments. (**B**) Pan-drug p53-independent signature. Only one gene was upregulated by all drugs independent of p53. (**C**) Pan-drug p53-dependent signature. Most genes regulated by all drugs were regulated dependent of p53.

A total of 961 transcripts were significantly up- or down-regulated (fold change ≥2 or ≤−2, *p*-value < 0.05) by at least one drug and principal component analysis (PCA) mapping demonstrated clear separation between wild-type and p53−/− cells (Supplementary Figure 4A, 4B). These 961 genes (the “master list”) were divided into pan-drug, drug-specific, or drug class-specific signatures which were further divided into p53-independent and -dependent signatures (Figure 2B). Each of these complete signatures, along with additional information for each gene in the master list (Probeset IDs, statistics, descriptions, etc.) can be found in Supplementary Table 1. The filtering method for inclusion in p53-indepenent and -dependent signatures is illustrated in Figure 2C. Not surprisingly, the transcriptomic response to each of the drugs varied greatly with only 36 transcripts being upregulated or downregulated across all drug treatments (Figure 3A). The pan-drug signature was divided into p53-independent and p53-dependent signatures (Figure 3B and 3C, respectively) which revealed that the majority of the pan-drug transcriptomic response was p53-dependent and supporting the idea that p53 is a master regulator of the cellular response to chemotherapy (Figure 3C).

As genes within this pan-drug signature were regulated across drugs with various primary targets and mechanisms of action, it is likely that they are fundamental to the response to chemotherapy, thus this signature may provide a set of prognostic biomarkers predicting response to chemotherapy. By identifying fundamental elements of the p53 program in CRC, this signature may also be used to more logically direct the development of p53-reactivating compounds specifically for treatment of CRC. It is notable that 10 of the transcripts within the pan-drug signature were classified as “identified by AceView”, meaning that they have unknown function and in some cases have unknown coding potential. One of these transcripts, skargluby, appeared at the top of the list. After splicing, skargluby is antisense to 457 base pairs of CDKN1A (coding for p21^WAF1^), raising the possibility of regulated alternate expression. These findings suggest that much remains unknown regarding the cellular response to chemotherapy used in clinical treatment of CRC.

B-cell translocation gene 2 (BTG2) was the most highly induced gene in the 5-FU, cisplatin, and oxaliplatin p53-dependent gene signatures and the second most highly induced gene in the CPT-11 p53-depenedent signature (Figure 3C). BTG2 is a tumor suppressor that plays a role in the p53-dependent component of the DNA damage response and its low expression correlates with more severe disease in breast and prostate cancer [26], so it is likely that BTG2 plays an important role in contributing efficacy of these drugs for treatment of colorectal cancer. GDF15, or growth differentiation factor 15, was also highly induced across all drug treatments (Figure 3C). This TGF-beta ligand likely promotes epithelial to mesenchymal transition and metastasis in CRC through activation of Smad2 and Smad3 pathways. Evaluation of GDF15 in patients in several studies revealed that high levels correlate with increased chances of metastasis, lower overall survival, and weight loss. GDF15 neutralization in non-human primates decreased levels of cisplatin-induced weight [27–29]. Conversely, 5-FU-resistant CRC cells express lower levels of GDF15 compared to 5-FU-sensitive cells and transient expression of GDF15 restores sensitivity, suggesting that this gene plays an important role in 5-FU-mediated cell death [30]. Therefore, GDF15 likely plays a multiplex role in the response to chemotherapy and careful investigation is needed before combination therapies are considered.

Further investigation of other transcripts in the pan-drug signature reveal that most have a known tumor suppressive function but others such as PRDM1 [31, 32], SESN1/2 [33, 34] may play a complicated or multiplex role in CRC and other cancers. Together, these results indicate that the p53 response to chemotherapy includes genes that likely contribute to efficacy and toxicity, and may contain transcripts that counteract anti-tumor effects of the drugs.

### Drug-specific signatures suggest novel mechanisms of efficacy and toxicity specific to 5-FU, CPT-11, oxaliplatin, or cisplatin

**Figure 4:**
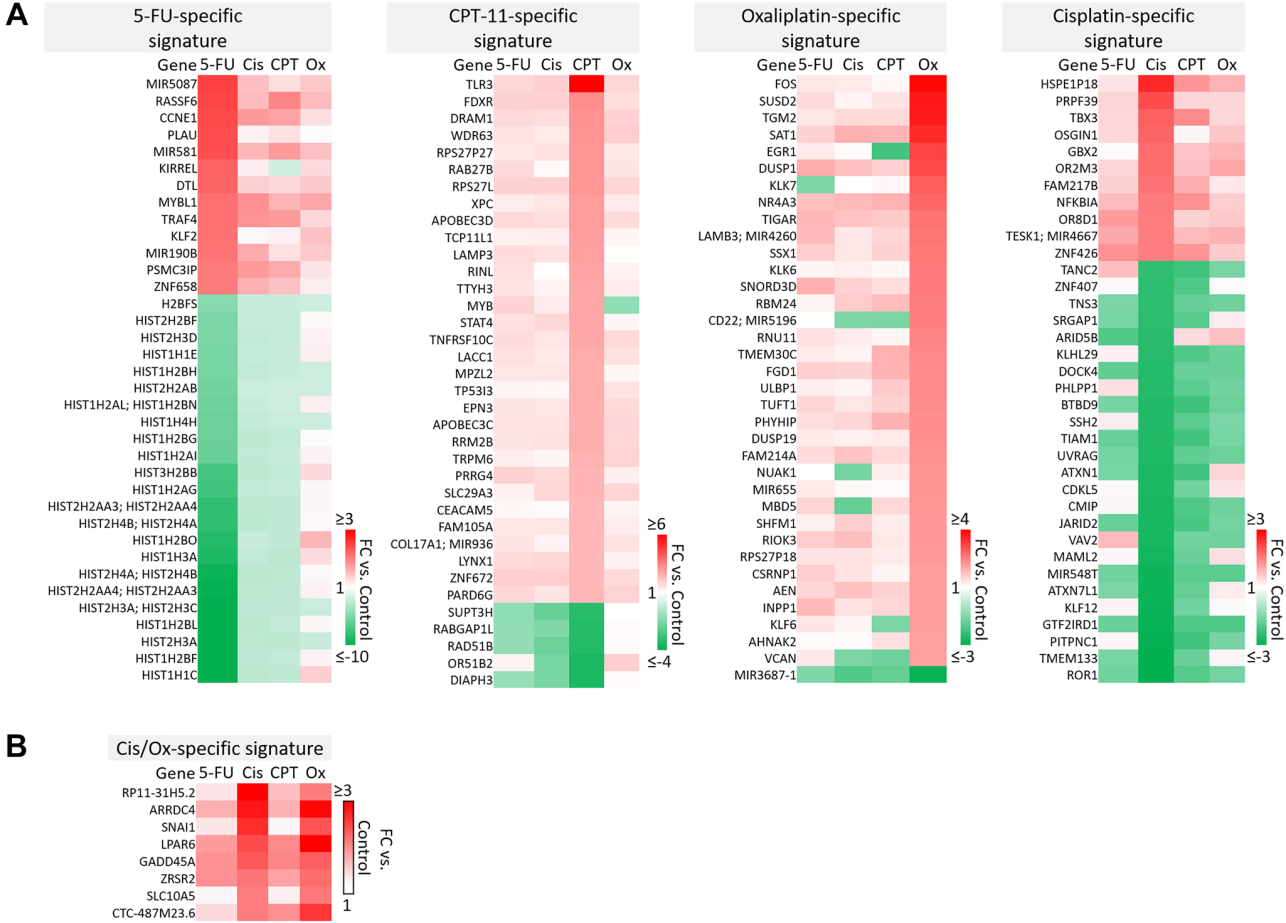
Drug-specific gene signatures after 5-FU, CPT-11, oxaliplatin, and cisplatin treatment of human colorectal cancer cells. (**A**) The most strikingly unique transcripts in each drug-specific signature are shown. Complete drug-specific signatures can be found in Supplementary Table 1. (**B**) Eight transcripts were regulated uniquely by platinum-based compounds.

**Figure 5:**
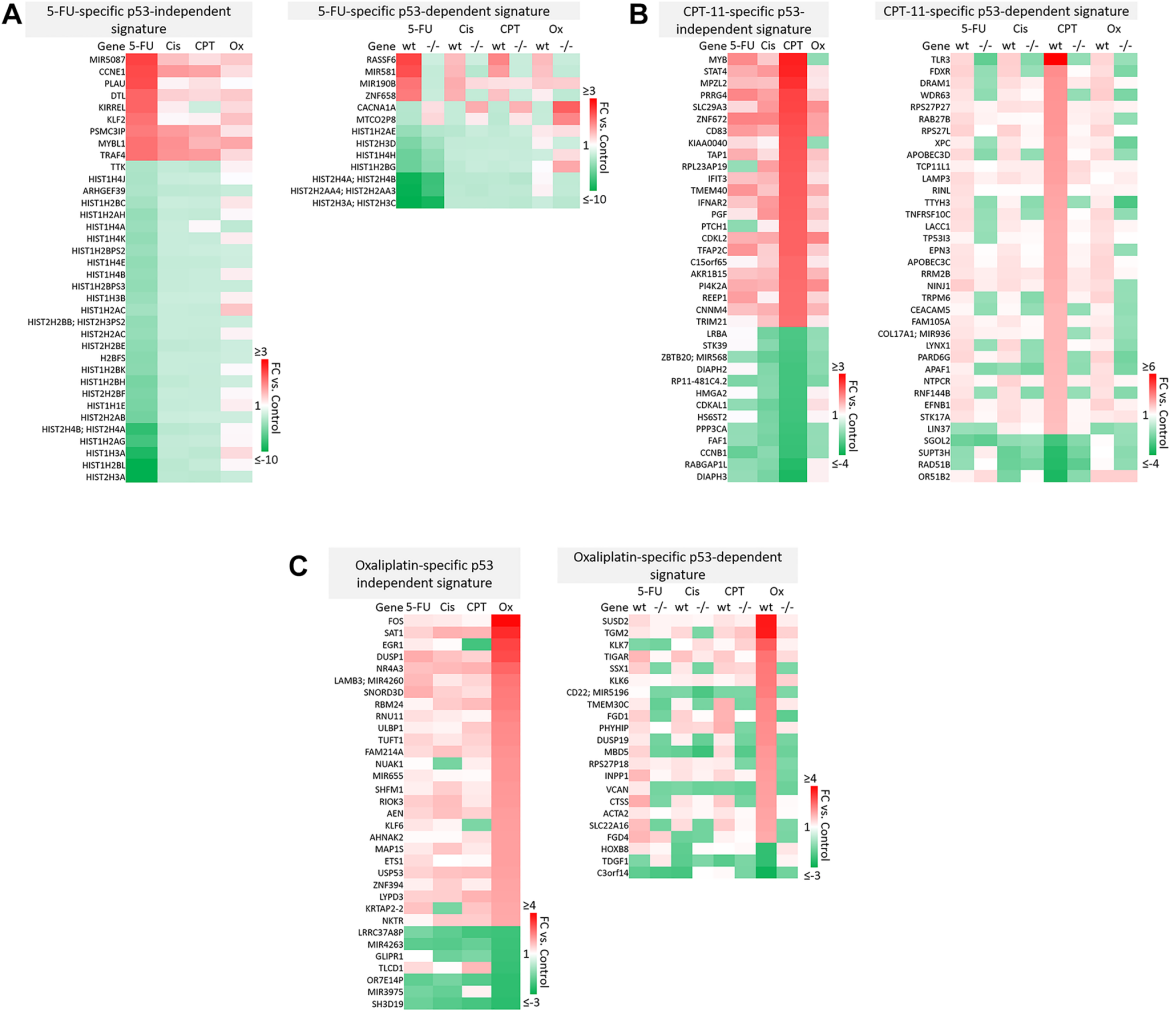
Drug-specific gene signatures divided into p53-independent and p53-dependent subsets after 5-FU, CPT-11, oxaliplatin, and cisplatin treatment of human colorectal cancer cells. Drug-specific signatures from Figure 4 were divided into p53-independent and p53-dependent subsets using the filtering method in Figure 2C. Drug-specific effects of (**A**) 5-FU, (**B**) CPT-11, and (**C**) oxaliplatin are shown.

**Figure 6:**
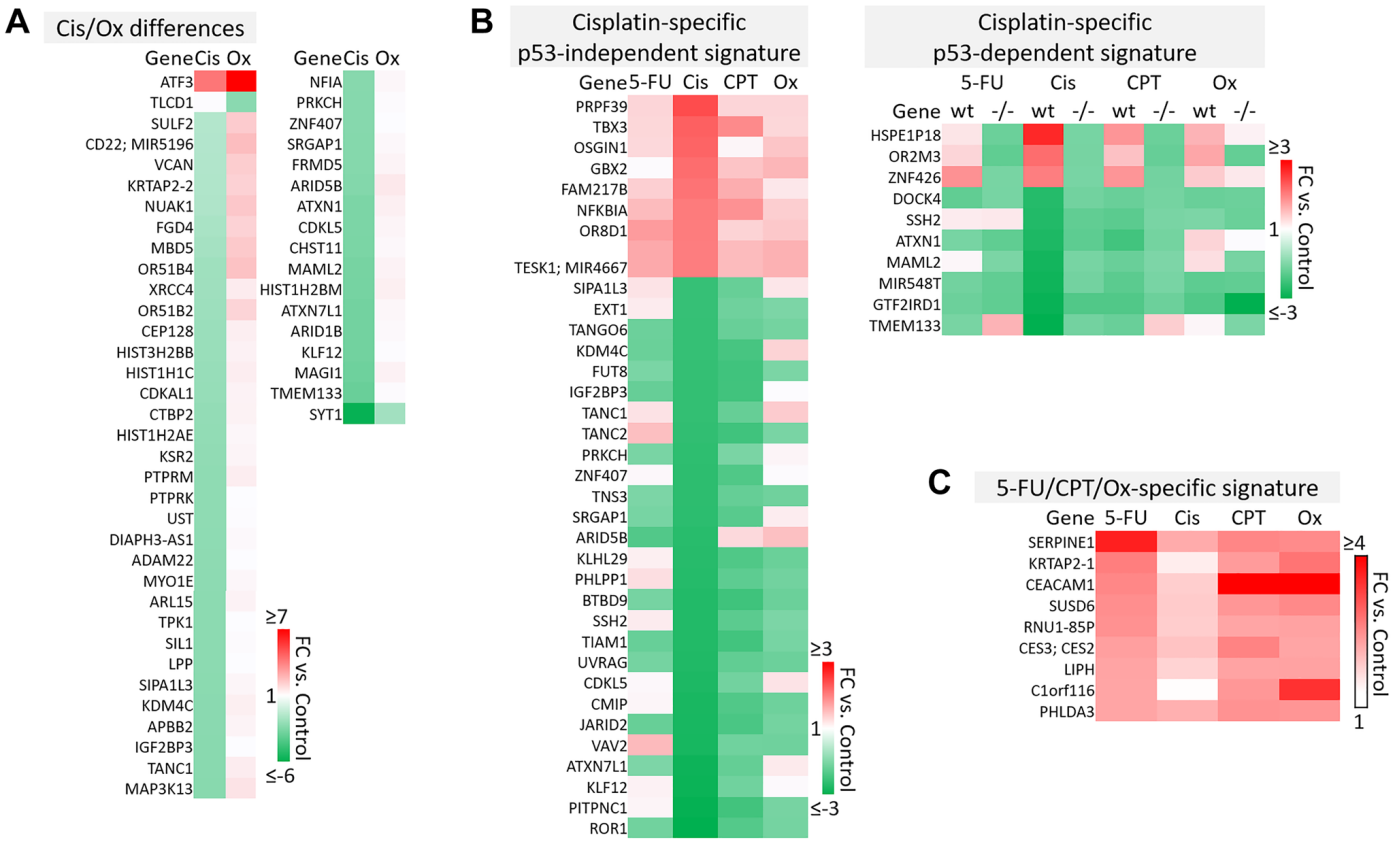
Cisplatin-specific and 5-FU/CPT-11/oxaliplatin-specific gene signatures. (**A**) Transcripts that are differentially regulated by cisplatin and oxaliplatin. (**B**) Cisplatin-specific gene signature divided into p53-independent and p53-dependent subsets. (**C**) 5-FU/CPT-11/oxaliplatin-specific signature contains transcripts that are upregulated by 5-FU, CPT-11, and oxaliplatin but are unaffected by cisplatin.

A majority of genes were regulated by 5-FU, CPT-11, oxaliplatin, and cisplatin in a drug-specific manner. The most striking differences from these signatures are shown in Figure 4A–4B. Complete drug-specific signatures can be found in Supplementary Table 1. Drug-specific signatures were further divided into p53-independent and p53-dependent signatures (Figure 5A–5C, Figure 6B). Both p53-independent and p53-dependent signatures revealed novel drug-specific effects that may indicate drug-specific mechanisms of efficacy.

Notable findings include a strikingly unique down-regulation of histone genes by 5-FU both in a p53-independent and -dependent manner (Figure 5A). This finding is supported by previous studies which demonstrated that ionizing radiation-mediated DNA damage induces down-regulation of histone genes through the G1 checkpoint pathway, and that histone H2 levels can be regulated by 5-FU. As the upregulation of histone proteins is needed in every round of cell replication, the downregulation of these genes by 5-FU may be a novel mechanism of efficacy of this drug. This mechanism, and whether or not it is 5-FU-specific, has yet to be investigated [35, 36].

Also interesting was the p53-dependent, CPT-11-specific regulation of transcripts including TLR3, a toll-like receptor known to promote anti-cancer immunity through activation of type I IFN; FDXR, or ferredoxin reductase, whose interaction with p53 is critical for tumor suppression via iron homeostasis; and DRAM1, a p53 target gene that modulates autophagy and apoptosis [37–39] (Figure 5B). Oxaliplatin uniquely upregulated SUSD2, which is commonly downregulated in CRC and interacts with the potential novel cytokine CSBF/C10orf99 to inhibit CRC cell growth, and SAT1, whose levels are also lower in patients with cancer and plays a critical role in ferroptosis [40, 41] (Figure 5C).

p53-independent and p53-dependent signatures also revealed novel drug-specific effects that may indicate drug-specific mechanisms of toxicity. For example, FOS, a commonly used marker of neuronal damage, was uniquely upregulated by oxaliplatin and thus could play a role in oxaliplatin-induced peripheral neuropathy. Others have reported that oxaliplatin upregulates FOS in neurons *in vitro*, but whether this effect is unique to oxaliplatin remains to be investigated. Interestingly, another group reported that oxaliplatin-treated mice exhibited neuronal damage (demonstrated by an upregulation of FOS and ATF3) that was reversible by treatment with metformin, which they suggest as a possible combination therapy to prevent/treat this side effect [42, 43]. Accordingly, ATF3 appeared in a list of transcripts which were regulated differentially by cisplatin vs. oxaliplatin (Figure 6A). Further analysis is needed to determine if drug-specific regulation of transcripts is relevant at later time points.

The dataset presented here may also be able to contribute to our understanding of why cisplatin has failed in the treatment of CRC patients, while similar platinum-based compounds like oxaliplatin have efficacy [44]. While both these drugs rely on creation of adducts to halt DNA synthesis and repair, less adducts are needed for oxaliplatin to have a more potent effect, suggesting other mechanisms are at play [14]. Uncovering cisplatin-specific mechanisms may identify a set of transcripts that are not likely to contribute to efficacy in CRC patients, and may actually lead to additional unnecessary toxicity (Figure 6B). These cisplatin-specific mechanisms include upregulation of PRPF39, a pre-mRNA processing factor known to play a key role in sensitivity to cisplatin [45], and downregulation of PITPNC1, which promotes metastasis-associated vesicular secretion [46]. Curiously, cisplatin also downregulated ROR1, whose high expression correlates with worse overall survival in CRC patients [47].

The lack of efficacy of cisplatin in CRC despite these seemingly positive effects may be explained by other transcripts in the cisplatin-specific signature that have tumor-promoting function. These include TBX3, which promotes epithelial to mesenchymal transition and predicts poor prognosis in colorectal cancer [48], and GBX2, which promotes growth of breast and prostate cancer cells [49, 50]. Together, these findings indicate that while cisplatin contributes to an anti-cancer response in CRC cells, these mechanisms may be largely counteracted by other cancer-promoting transcripts that may play a particularly significant role in CRC (Figure 6B).

A signature containing genes that are upregulated by 5-FU, CPT-11, and oxaliplatin but that were unaffected by cisplatin may suggest additional reasons for differences in efficacy profiles across these drugs (Figure 6C). Interestingly, some transcripts in this signature may be biomarkers for worse outcomes (SERPINE1) or play a dichotomous role in CRC (CEACAM1) [51–53]. However C1orf116, which was discovered in 2017 as a driver of epithelial phenotype in epithelial-to-mesenchymal transition, also appeared in this 5-FU/CPT-11/oxaliplatin-specific signature [54] and may contribute to their unique efficacy (Figure 6C). It is likely that a complex interplay of the transcripts within cisplatin-specific and 5-FU/CPT-11/oxaliplatin-specific signatures might explain the lack of cisplatin efficacy in treatment of CRC. The data here identify several candidate transcripts that play into these mechanisms. Further investigation and validation could enhance our understanding of gene signatures that are especially important for an effective response to chemotherapy in CRC.

### Subsets of transcriptomic signatures in response to 5-FU, CPT-11, oxaliplatin, and cisplatin correlate with patient outcomes

**Figure 7:**
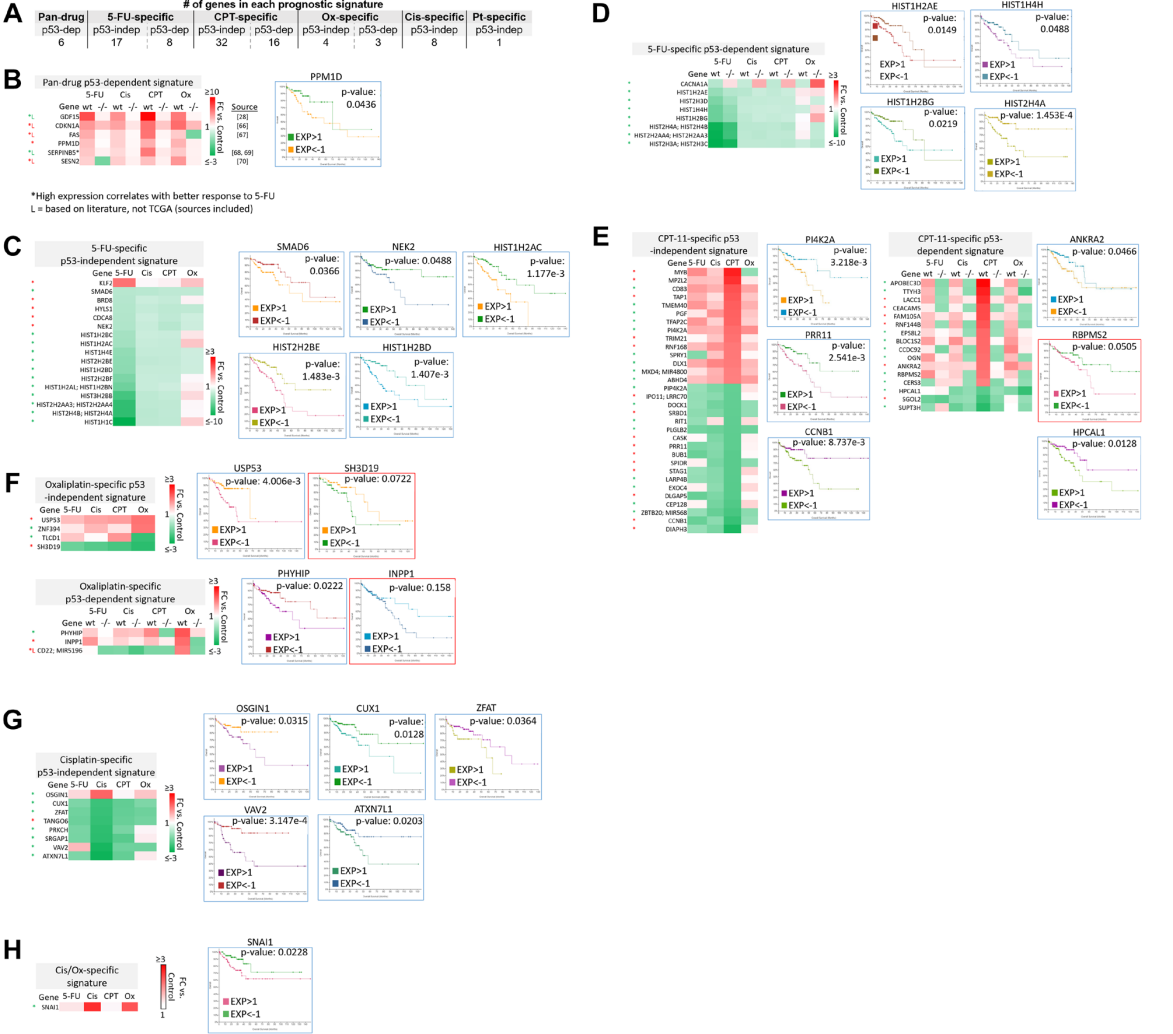
Subsets of transcriptomic signatures in response to 5-FU, CPT-11, oxaliplatin, and cisplatin correlate with patient outcomes. TCGA (which contains data on basal gene expression) and literature searching was used to establish subsets of gene signatures that correlate with patient outcomes. A logrank test *p*-value under 0.05 was considered significant, while a *p*-value between 0.05–0.2 was considered of potential interest. Both of these were included in the prognostic signatures. (**A**) The total number of prognostic transcripts in each gene signature is shown. Pt = platinum-based drug. (**B**–**H**) Genes within each signature that correlate with patient outcomes are listed along with representative Kaplan-Meier curves. Blue border = Logrank test *p*-value < 0.05. Red border = Logrank test *p*-value between 0.05–0.2. Red (^*^) = high expression correlates with better overall survival. Green (^*^) = low expression correlates with better overall survival [28, 66–70].

The clinical relevance of these signatures was evaluated with The Cancer Genome Atlas (TCGA), which contains RNA-sequencing and microarray data on patient samples prior to treatment. Overall survival of colorectal adenocarcinoma patients was correlated with high vs. low (>1 or <−1 standard deviation from the mean, respectively) basal expression of transcripts within pan-drug, drug-specific, drug class-specific, p53-independent, and p53-dependent signatures. High vs. low basal expression of transcripts in the p53-dependent signatures were evaluated separately in p53 wild-type and p53 mutated patient groups, though this separation did not have a significant effect on correlation with overall survival. Existing literature was evaluated to supplement TCGA data in the establishment of these signatures. Nearly all signatures contained transcripts that significantly correlated with overall survival (Figure 7A).

The identity of these transcripts and representative Kaplan-Meier curves are shown in Figure 7B–7H. Additional Kaplan-Meier curves are shown in Supplementary Figure 5. Most notably, low basal expression of 17 histone genes that were uniquely downregulated by 5-FU correlated with improved overall survival (Figure 7C–7D, Supplementary Figure 5). Only two histone genes that were downregulated by 5-FU correlated with improved survival when highly expressed (Supplementary Figure 5, grey border). Interestingly, many transcripts whose high expression correlated with improved survival were downregulated by the drugs, and vice versa (Figure 7B–7H). These could be mechanisms by which the drugs contribute to toxicity or resistance, suggesting that potential combination treatments to limit these effects should be investigated. Together, these signatures can serve as novel prognostic biomarkers for CRC patients that are personalized based on p53 status.

### Cytokines TRAILR2, IL-8, VEGF, and ferritin are regulated differently across treatments with 5-FU, CPT-11, oxaliplatin, cisplatin, and clinically relevant combinations

It is well-documented that chemotherapy increases amounts of circulating cytokines [55–57]. Optimizing this induction both in terms of identity and magnitude is critical for maximizing the anti-tumor immune effects of chemotherapy and avoiding cytokine storm [58]. Some work has been done to evaluate the effects of 5-FU, CPT-11, oxaliplatin, and cisplatin on cytokine levels [57, 59, 60], but none have compared directly across all four drugs, clinically relevant combinations, and p53 status.

**Figure 8:**
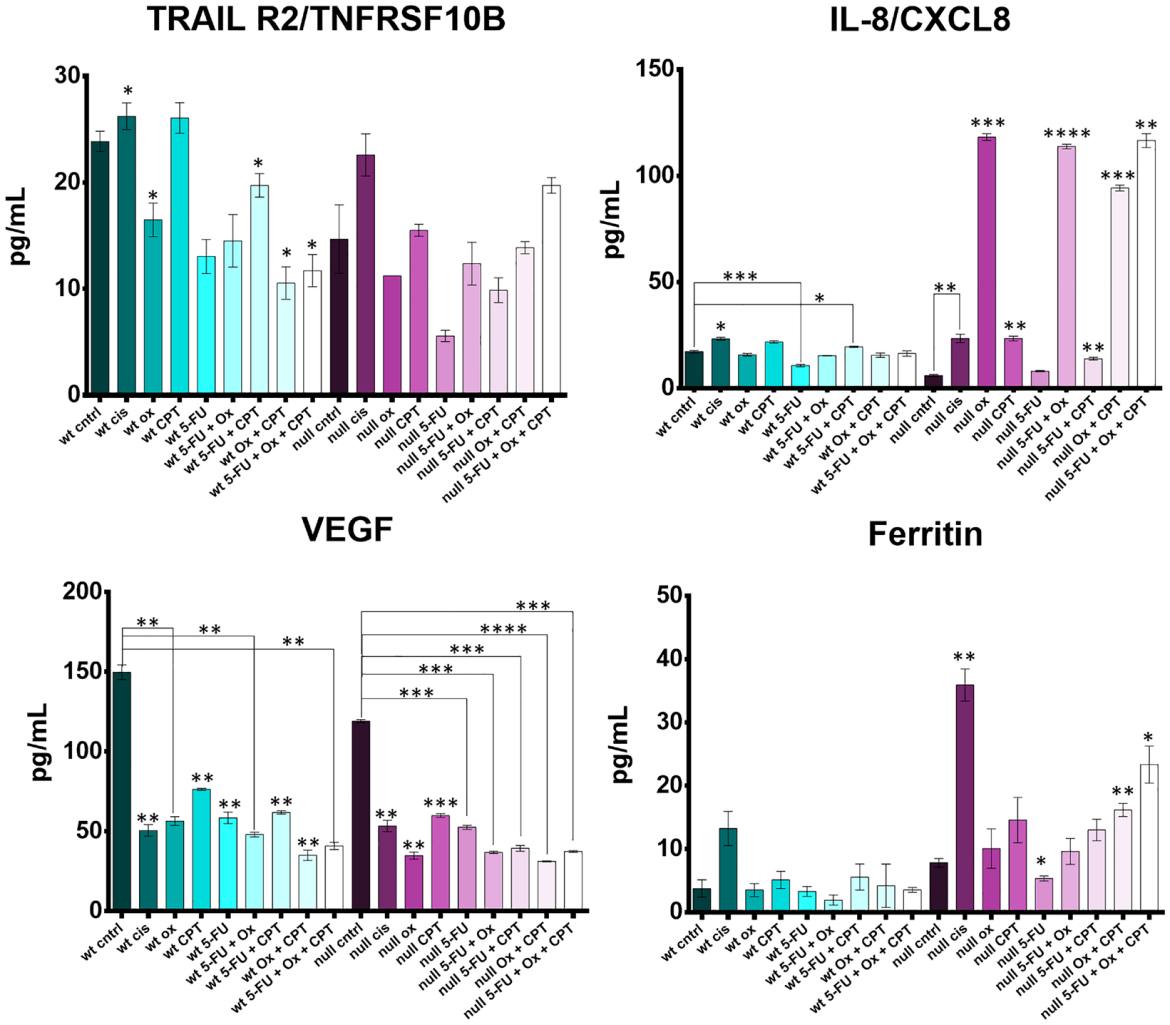
Cytokine profiling reveals drug- and drug combination-specific induction of TRAILR2, IL-8, VEGF, and ferritin after 5-FU, CPT-11, oxaliplatin, and cisplatin treatment of human colorectal cancer cells. HCT116 and HCT116 p53−/− cells were treated at with the four drugs at their IC50s and combination treatment groups received 2–3 drugs, each at their individual IC50 concentration for 48 hours. Cytokine levels in the cell supernatants were measured with the Luminex 200 platform and significant differences (*p*-value < 0.05 by one-way ANOVA) between control and treated groups was calculated.

HCT116 and HCT116 p53−/− cells were treated with the four drugs 5-FU, CPT-11, oxaliplatin, and cisplatin at their IC50s and combination treatment groups received 2–3 drugs, each at their individual IC50 concentration. Cytokine levels in the cell supernatants were measured with the Luminex 200 platform and significant differences between control and treated groups were noted for cytokines TRAILR2, IL-8, VEGF, and ferritin (Figure 8).

Soluble TRAILR2 (death receptor 5; DR5) is a decoy receptor for TRAIL, an apoptosis-inducing cytokine. TRAILR2 was down regulated by oxaliplatin and 5-FU, was not affected by CPT-11, and was increased by cisplatin. TRAILR2 levels were lower in the oxaliplatin and 5-FU treated cells (effect was not synergistic) compared to cisplatin and CPT-11 treated cells. Downregulation of TRAILR2 by oxaliplatin seemed to be dependent on p53, but p53-independent by 5-FU. Cisplatin upregulated TRAILR2 irrespective of p53 status, suggesting a conflicting mechanism between cisplatin, oxaliplatin, and 5-FU which has not been reported previously.

Upregulation of IL-8, a pro-inflammatory cytokine thought to have an immunosuppressive effect in the tumor microenvironment and whose levels in serum correlate with CRC progression, was drastically different across p53 status [61, 62]. Most notably, oxaliplatin had no effect on IL-8 levels in the wild-type cells, but induced IL-8 the p53−/− cells. This finding could have a significant impact on how oxaliplatin is administered to patients based on their p53 status. CPT-11 single agent treatment and CPT-11 + 5-FU treatment also caused a moderate increase in IL-8 irrespective of p53 status.

VEGF, or vascular endothelial growth factor, is a pro-angiogenic cytokine responsible for supplying oxygen and nutrients to the tumor as well as promoting cancer cell escape. All four drugs significantly downregulated release of VEGF by both HCT116 and HCT116−/− cells with no apparent synergistic effect of drug combinations.

Ferritin is known to have several tumor-promoting effects including protection of cancer cells from reactive oxygen species and promoting the pro-tumorigenic M2 program in macrophages [63], and is used as a prognostic marker in some cancers [64]. Increased ferritin expression also limits ferroptosis, an iron-dependent form of cell death distinct from apoptosis [65]. In WT cells, no drug significantly affected ferritin levels. In p53−/− cells, there was a large increase in ferritin after cisplatin treatment. Interestingly, oxaliplatin plus CPT-11 and 5-FU plus oxaliplatin and CPT-11 treatment increased ferritin levels more so than single treatments, though this effect does not seem to be synergistic. No notable changes were observed in GM-CSF, C-reactive protein, CXCL13, IL-18, CCL22, or IFN-alpha profiles (Supplementary Figure 6).

Together, these data suggest that chemotherapeutics and p53 status can have a large impact on cytokine regulation. Most notable findings include an increase in IL-8 by oxaliplatin and increase in ferritin by cisplatin. As both of these cytokines are thought to contribute to cancer cell survival, care should be taken in administering specific therapies to patients based on their basal expression of these cytokines and the p53 status of the tumors.

## DISCUSSION

Here, we describe a database of transcriptomic and cytokine responses of HCT116 and HCT116 p53−/− cells to clinically relevant chemotherapeutics used to treat CRC including 5-FU, CPT-11, oxaliplatin, and cisplatin (not used to treat CRC but included for comparison to oxaliplatin). Our direct analysis across drug treatments and across p53 status revealed several novel drug-specific mechanisms of efficacy and toxicity. These include downregulation of histone genes by 5-FU (that significantly correlates with improved survival in CRC patients) and upregulation of FOS and ATF3 by oxaliplatin (which may contribute to peripheral neuropathy). Besides a number of other novel drug-specific effects, BTG2 was identified as a top gene upregulated by all four drugs, suggesting its critical role in the cellular response to chemotherapy in CRC.

Several transcripts whose high expression correlated with improved survival were downregulated by the drugs suggesting potential mechanisms by which the drugs may contribute to toxicity or resistance. In the future, combination treatments to limit these effects should be further investigated.

BTG2 was identified as a top gene upregulated by all four drugs (5-FU, CPT-11, oxaliplatin, and cisplatin). This upregulation of BTG2 by compounds with vastly different mechanisms and p53 induction profiles emphasizes its importance in the cellular response to DNA damage and reveals its critical role in mediating the p53 response to chemotherapy used to treat CRC. As BTG2 upregulation is p53-dependent, particular focus should be dedicated to this transcript when designing p53-reactivating therapies and for further analysis of relationship to drug response and outcomes in patient cohorts with colorectal cancer. Skargluby, which after splicing encodes a transcript antisense to gene CDKN1A, is upregulated by chemotherapeutics used to treat CRC and requires further investigation regarding its impact on drug sensitivity and patient outcomes.

Soluble TRAILR2 (death receptor 5; DR5) is a decoy receptor for TRAIL, an apoptosis-inducing cytokine. TRAILR2 was down-regulated by oxaliplatin and 5-FU, was not affected by CPT-11, and was increased by cisplatin. Soluble TRAILR2 may provide a readout on a mechanism by which certain tumors may evade the innate immune system. There was an increase in IL-8 by oxaliplatin and increase in ferritin by cisplatin which may contribute to cancer cell survival. It is of clinical interest that 5-FU, CPT-11, and oxaliplatin (as well as cisplatin) downregulated VEGF production by treatment of CRC cells. Further studies could further investigate the impact of VEGF downregulation on bevacizumab efficacy as the drugs used to treat CRC are often combined with bevacizumab.

Other future directions include evaluating outcomes in groups of patients who have received one or more of the chemotherapy drugs. This type of analysis is necessary to answer important questions such as whether induction of the transcripts during chemotherapy treatment predicts long-term survival, and whether high versus low basal expression of these transcripts in patients reduces efficacy of specific drugs. While these questions may be partially answered with only basal expression data, they cannot be answered definitively without information on treatments received. Additionally, later time points (>8 hours) should be evaluated due to our initial finding that variability in the p53 response (as evaluated by upregulation of p21 and DR5 via western blot) did not remain consistent over time. Later time points should also be evaluated because work by others indicates that some unique transcripts identified here (for example, SUSD2 and SAT1 by oxaliplatin) are upregulated >2 fold change by the other drugs at later time points. Despite this, the magnitude of difference in fold change of these transcripts across the drug treatments may suggest that there are biologically relevant effects at early time points.

This is the first dataset to directly compare transcriptomic and cytokine responses of CRC cells to equitoxic doses of 5-FU, CPT-11, oxaliplatin, and cisplatin across p53 status, and thus is the first to reveal vast differences in the magnitude of fold change of several genes and cytokines across the drug treatment groups. This dataset also permits evaluation of gene and cytokine responses that are similar across drugs, emphasizing or revealing their critical role in the p53-independent or p53-dependent cellular responses to chemotherapy in CRC. Together, this work furthers our understanding of the mechanisms that mediate efficacy and toxicity in the treatment of CRC, providing guidance in combination treatment selection, insights for development of targeted therapies, and prognostic markers for CRC patients based on the treatments they receive. Further investigation of specific transcripts or sets of transcripts within these signatures can uncover additional mechanisms of pan-drug, drug-specific, drug class-specific, p53-independent, and p53-dependent efficacy and toxicity.

## MATERIALS AND METHODS

### Cell lines and culture conditions

HCT116 and HCT116 p53−/− human colorectal cancer cells (obtained as a gift from Bert Vogelstein, Johns Hopkins University, USA) were grown in McCoy’s 5A medium supplemented with 10% FBS and 1% penicillin/streptomycin at 37 degrees, 5% CO_2_. Cells were tested to ensure that they are mycoplasma free.

### Establishing IC50 doses

HCT116 and HCT116 p53−/− cells were treated with doses ranging from 0.08–80 μM of 5-FU, CPT-11, oxaliplatin, or cisplatin for 72 hours in a 96 well plate. Cell viability was measured using the CellTiterGlo assay (Promega G7570) and the IC50 dose was established from the resulting dose response curve.

### Western blots

A total of 5 × 10^5^ HCT116 and HCT116 p53−/− cells were plated in a 6-well plate and incubated for 12–16 hours before being treated with 5-FU, CPT-11, oxaliplatin, or cisplatin at their respective IC50 doses for several time points ranging from 1–48 hours. Proteins were extracted from cells with RIPA buffer containing protease inhibitor. Denaturing sample buffer was added, samples were boiled at 95 degrees for 10 minutes, and an equal amount of protein lysate was electrophoresed through 4–12% SDS-PAGE gels (Invitrogen) then transferred to PVDF membranes. The PVDF membrane was blocked with 5% non-fat milk (Sigma) in 1x TTBS. The primary antibodies indicated in the figures were incubated with the transferred PVDF membrane in blocking buffer at 4°C overnight. Antibody binding was detected on PVDF with appropriate Pierce HRP-conjugated secondary antibodies by the Syngene imaging system. Invitrogen Goat anti-Rabbit IgG (H + L) Secondary Antibody, HRP #31460 and Goat anti-Mouse IgG (H + L) Secondary Antibody, HRP #31430 were diluted 1:5000 in 2.5% non-fat milk.

### Microarrays

A total of 7 × 10^5^ HCT116 and HCT116 p53−/− cells were plated in a 6-well plate and incubated for 12–16 hours before being treated with 5-FU, CPT-11, oxaliplatin, or cisplatin at their respective IC50 doses for 8 hours. Cell pellets were collected and split into two tubes, one for western blot analysis and one for RNA extraction. Samples (30 in total) were randomized and RNA was isolated (Qiagen 74134) in batches of five to ensure high quality RNA product. Acceptable RNA concentration and quality were verified with Nanodrop and Bioanalyzer measurements. Clariom D Microarrays (ThermoFisher 902923) were conducted according to the manufacturer’s protocol in two batches on randomized samples to limit batch effects.

### TCGA analysis

The publicly available computational tool cBioPortal was used for analysis of TCGA data. Unless otherwise indicated, the Colorectal Adenocarcinoma PanCancer Atlas database (containing 592 total samples with RNA-seq data) was used. Differences in overall survival were evaluated between groups with high or low (>1 and <−1 standard deviation from the mean, respectively) basal expression of specific transcripts in the tumors irrespective of TP53 status and in patients with TP53 wild-type tumors only. Log-transformed mRNA expression z-scores were compared to the expression distribution of all samples. z-score threshold = ±2. A logrank test *p*-value under 0.05 (blue border in Supplementary Figure 5) was considered significant, while a *p*-value between 0.05–0.2 (red border) was considered of potential interest. Both of these were included in the prognostic signatures.

### Cytokine profiling

A total of 4 × 10^4^ HCT116 or HCT116 p53−/− cells were plated per well of a 24-well plate and incubated 12–16 hours before treatment with 5-FU, CPT-11, oxaliplatin, cisplatin, or various combination treatments at the appropriate IC50 (combination treatments received each drug at their individual IC50). Cell supernatants were collected at 48 hours after treatment and stored at −20^o^C. Samples were prepared and run in triplicate on a Luminex 200 Instrument (R&D LX200-XPON-RUO). Sample preparation was conducted and instrument settings were selected based on the Human Magnetix Luminex Assay (R&D LXSAHM) protocol.

### Statistical analysis

#### Microarrays

Applied Biosystems Transcriptomic Analysis Console (TAC) software was used to calculate fold-changes in gene expression relative to the untreated control cells. *p*-values <0.05 were considered significant.

#### Cytokine profiling

Results were analyzed in GraphPad prism and statistical significance across drugs was determined separately for WT and −/− cells with a one-way ANOVA. *p*-values <0.05 were considered significant.

## SUPPLEMENTARY MATERIALS




