# Supplementary material for: Pan-drug and drug-specific mechanisms of 5-FU, irinotecan (CPT-11), oxaliplatin, and cisplatin identified by comparison of transcriptomic and cytokine responses of colorectal cancer cells
Source: Oncotarget. 2021 Sep 28;12(20):2006–21. doi: 10.18632/oncotarget.28075 (PMC8487728; doi:10.18632/oncotarget.28075)
Supplement: Supplementary file 1 [file oncotarget-12-2006-s001.pdf]

Pan-drug and drug-specific mechanisms of 5-FU, irinotecan (CPT-11), oxaliplatin, and cisplatin identified by comparison of transcriptomic and cytokine responses of colorectal cancer cells

SUPPLEMENTARY MATERIALS

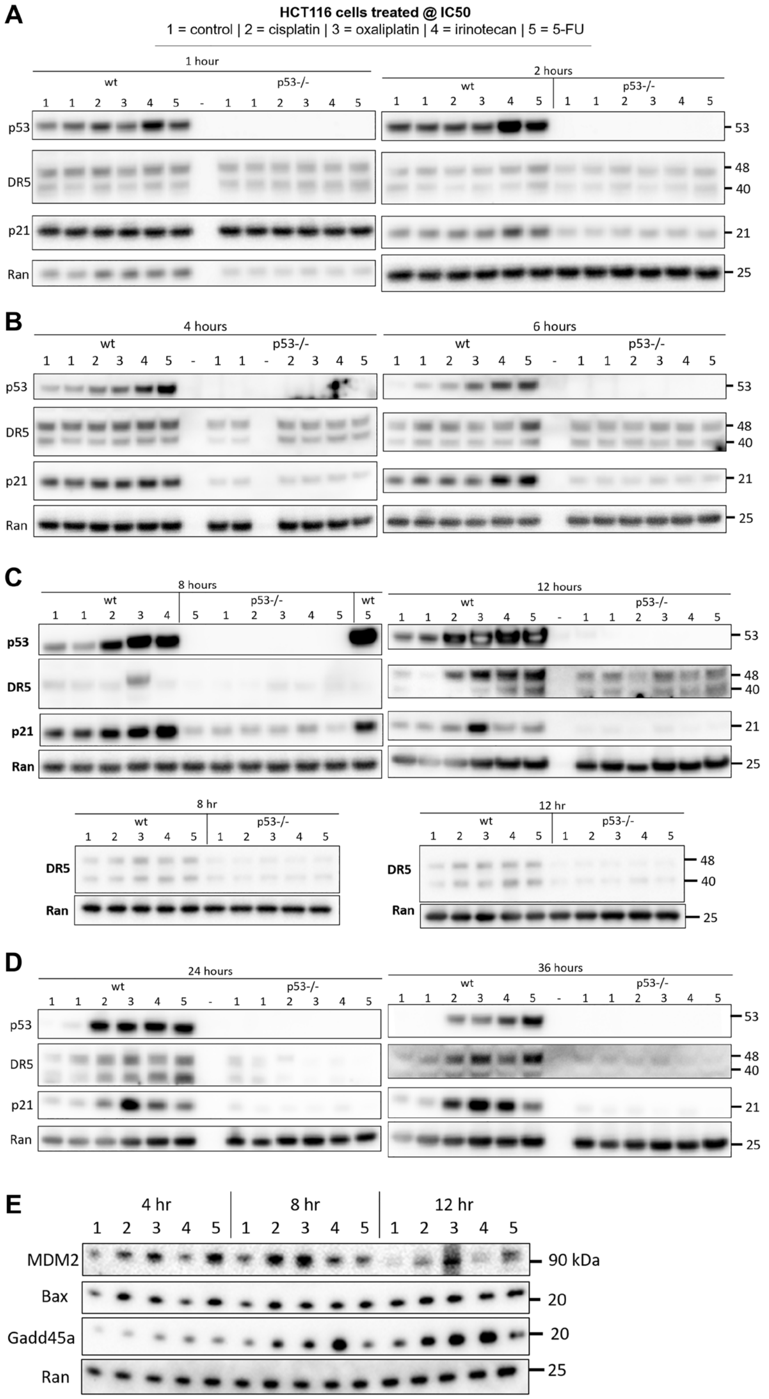

Supplementary Figure 1: Drug-specific variability in the kinetics of the p53 response to 5-FU, CPT-11, oxaliplatin, and cisplatin in human colorectal cancer cells. HCT116 and HCT116 p53−/− cells were treated with 5-FU, CPT-11, oxaliplatin and cisplatin at their respective IC50s for 1 hour, 2 hours (**A**), 4 hours, 6 hours (**B**), 8 hours, 12 hours (**C**), 24 hours, or 36 hours (**D**). Cells were similarly treated for 4, 8, or 12 hours in (**E**).

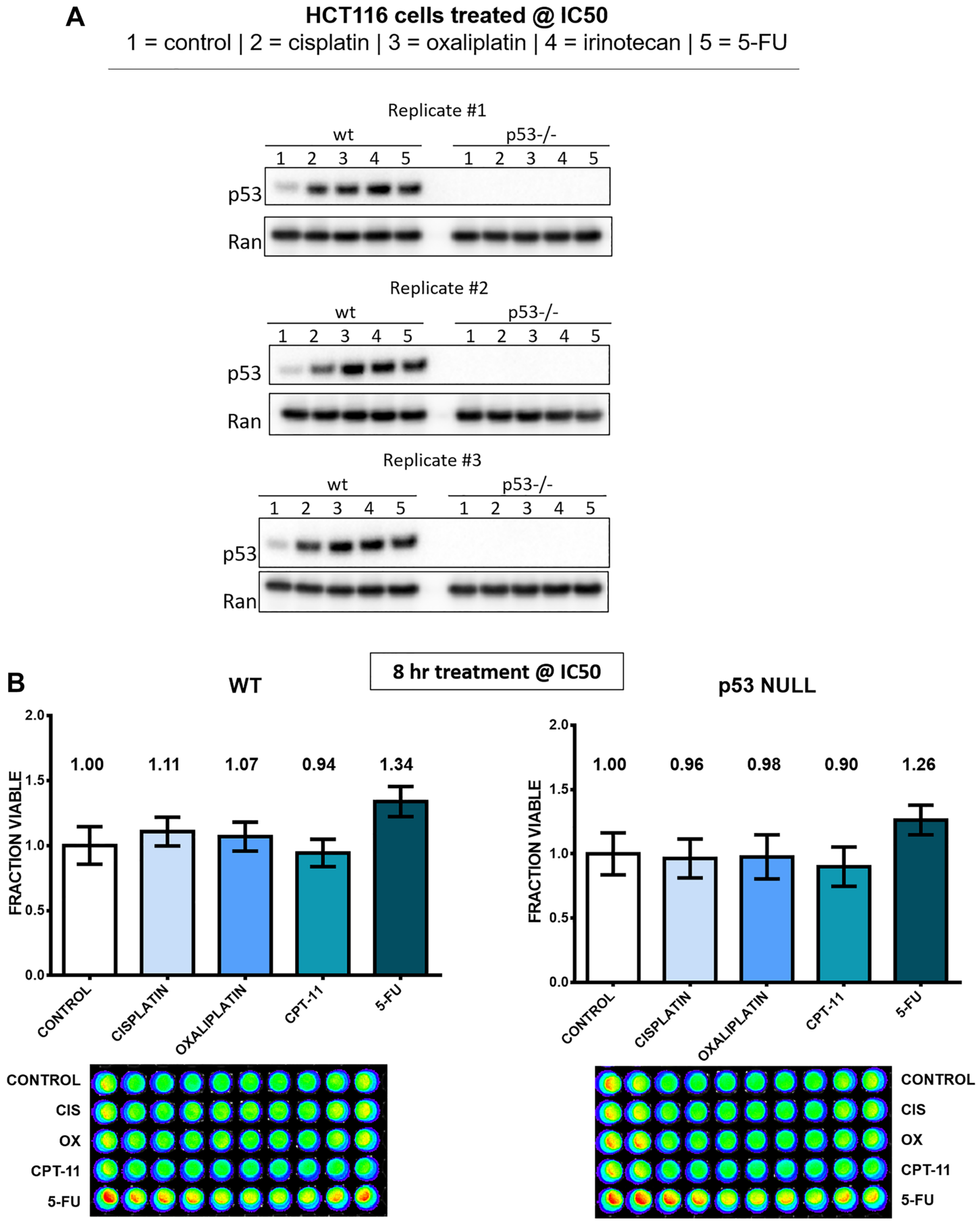

Supplementary Figure 2: Verification of p53 induction and equal cell death in samples used for microarray analysis. HCT116 and HCT116 p53−/− cells were treated with cisplatin, oxaliplatin, CPT-11, or 5-FU at their IC50 for 8 hours. (**A**) Western blot demonstrated that p53 was upregulated at this time point in all three biological replicates used for microarray analysis. (**B**) CellTiterGlo cell viability assay confirmed that there was no induction of cell death at this time point. Edge effect was noted for 5-FU treated cells but no cleavage of PARP was noted in western blots of similarly treated samples (Figure 1, 8 hr panel).

A

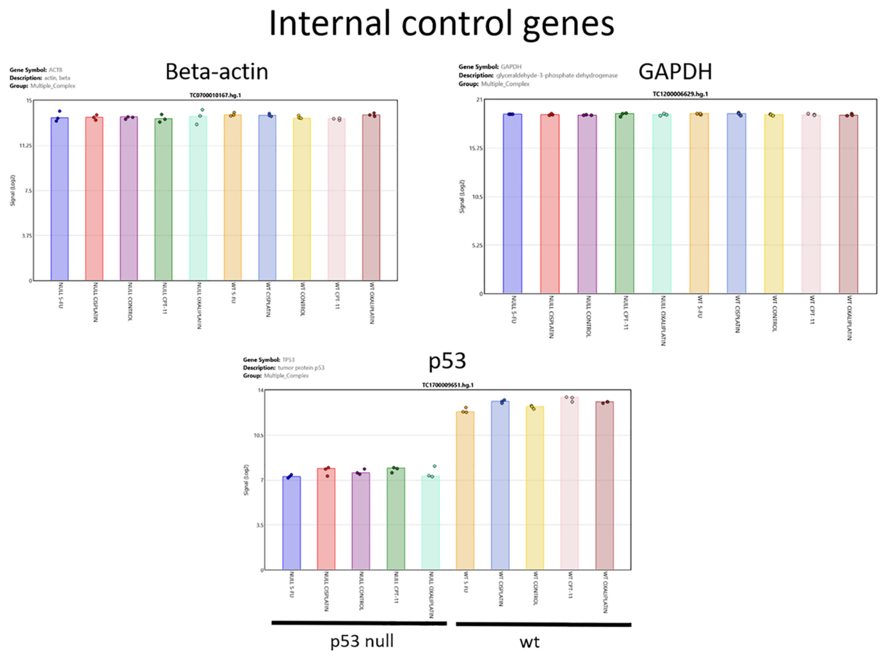

B Sample QC Overview

Sample #	Labeling Controls Threshold	Hybridization Controls Threshold	Pos vs Neg AUC Threshold	Condition
27	Out	Pass	Pass	NULL CONTROL
23	Out	Pass	Pass	NULL OXALIPLATIN
15	Out	Pass	Pass	NULL OXALIPLATIN
26	Out	Pass	Pass	WT CPT-11
28	Out	Pass	Pass	WT OXALIPLATIN
16	Pass	Pass	Pass	NULL 5-FU
14	Pass	Pass	Pass	NULL 5-FU
4	Pass	Pass	Pass	NULL 5-FU
25	Pass	Pass	Pass	NULL CISPLATIN
5	Pass	Pass	Pass	NULL CISPLATIN
1	Pass	Pass	Pass	NULL CISPLATIN
30	Pass	Pass	Pass	NULL CONTROL
22	Pass	Pass	Pass	NULL CONTROL
3	Pass	Pass	Pass	NULL CPT-11
29	Pass	Pass	Pass	NULL CPT-11
11	Pass	Pass	Pass	NULL CPT-11
21	Pass	Pass	Pass	NULL OXALIPLATIN
13	Pass	Pass	Pass	WT 5-FU
8	Pass	Pass	Pass	WT 5-FU
2	Pass	Pass	Pass	WT 5-FU
19	Pass	Pass	Pass	WT CISPLATIN
9	Pass	Pass	Pass	WT CISPLATIN
6	Pass	Pass	Pass	WT CISPLATIN
17	Pass	Pass	Pass	WT CONTROL
12	Pass	Pass	Pass	WT CONTROL
10	Pass	Pass	Pass	WT CONTROL
24	Pass	Pass	Pass	WT CPT-11
7	Pass	Pass	Pass	WT CPT-11
20	Pass	Pass	Pass	WT OXALIPLATIN
18	Pass	Pass	Pass	WT OXALIPLATIN

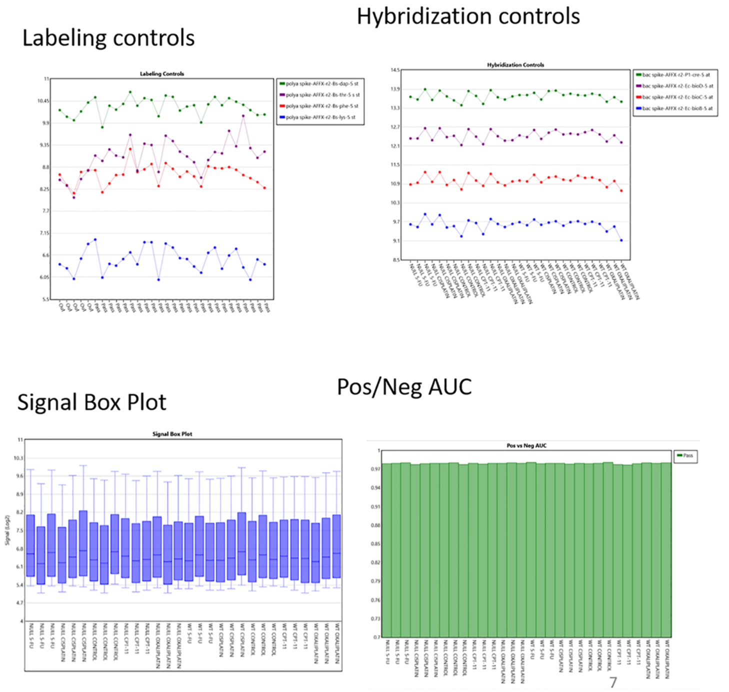

Supplementary Figure 3: Internal control genes in microarray analysis. (**A**) Housekeeping Beta-actin and GAPDH genes show no change across drug treatments and exhibit tightly clustered triplicates. p53 is lower in p53−/− cells, as expected. (**B**) Quality control was determined satisfactory for further analysis.

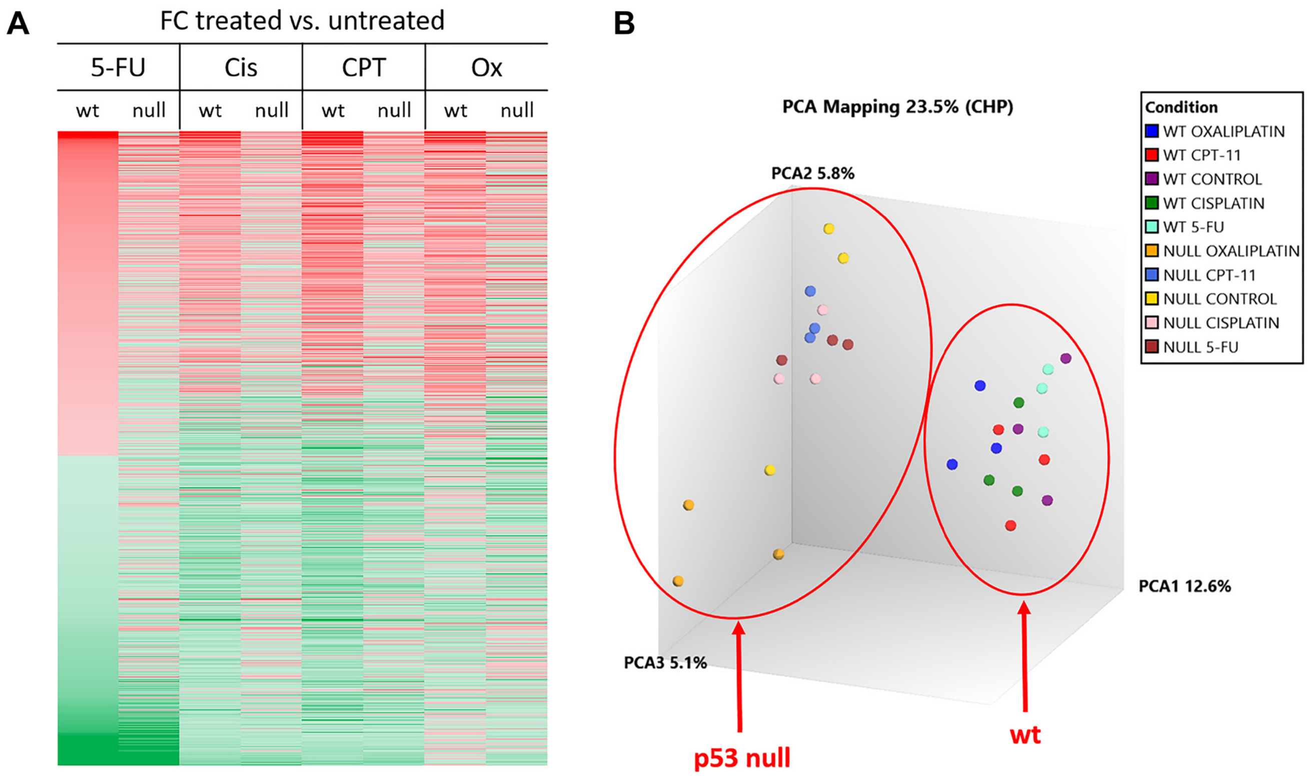

Supplementary Figure 4: Global view of gene expression across HCT116 & HCT116 p53−/− cells treated with 5-FU, cisplatin, CPT-11, or oxaliplatin. (**A**) The master list of transcripts regulated by at least one drug contained 961 genes. Regulation of the majority of these genes varied across drug and p53 status. (**B**) PCA mapping demonstrated clear separation of wild-type and p53−/− cells, as expected.

5-FU-specific signatures

p53-independent

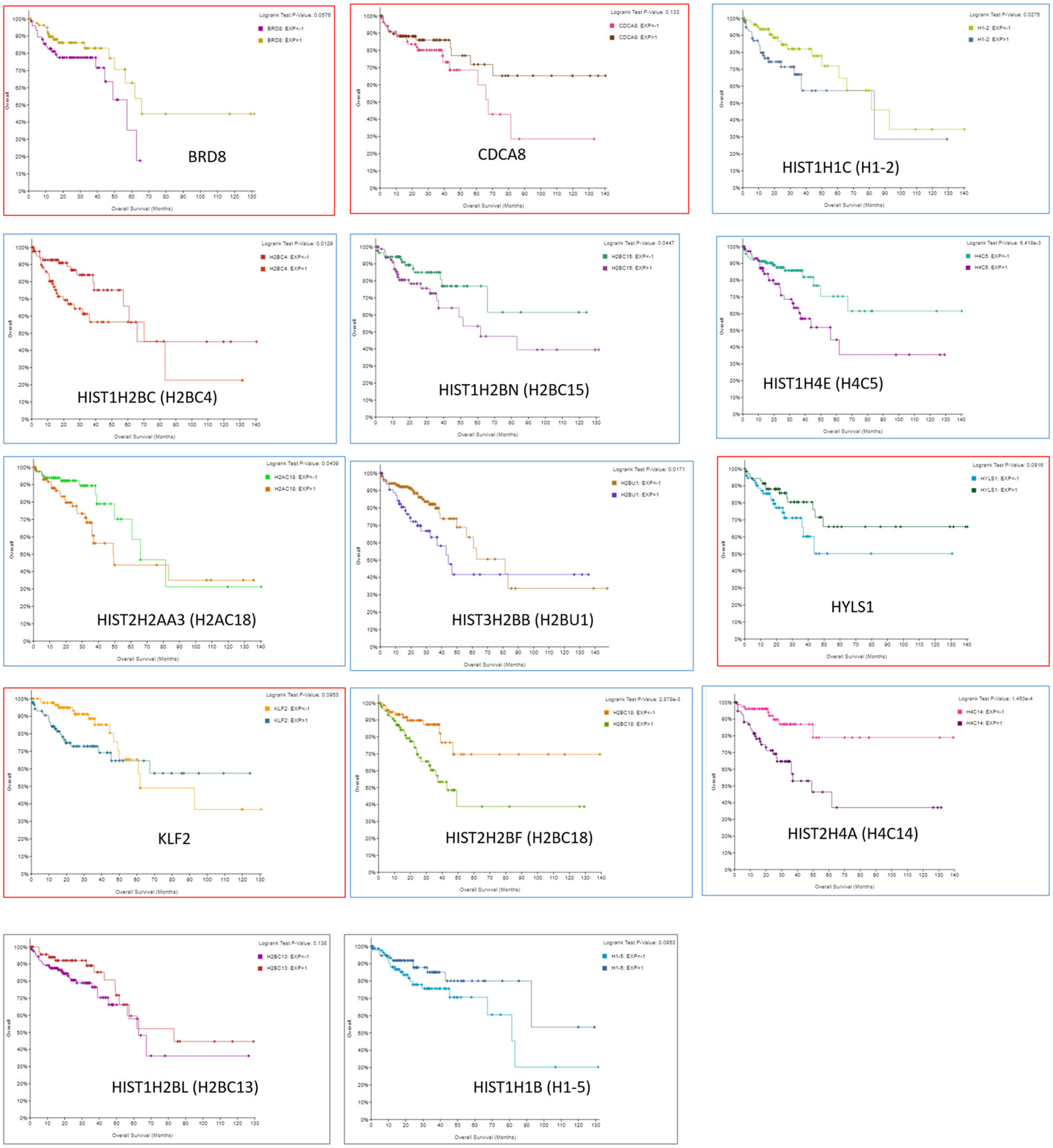

5-FU-specific signatures

p53-dependent

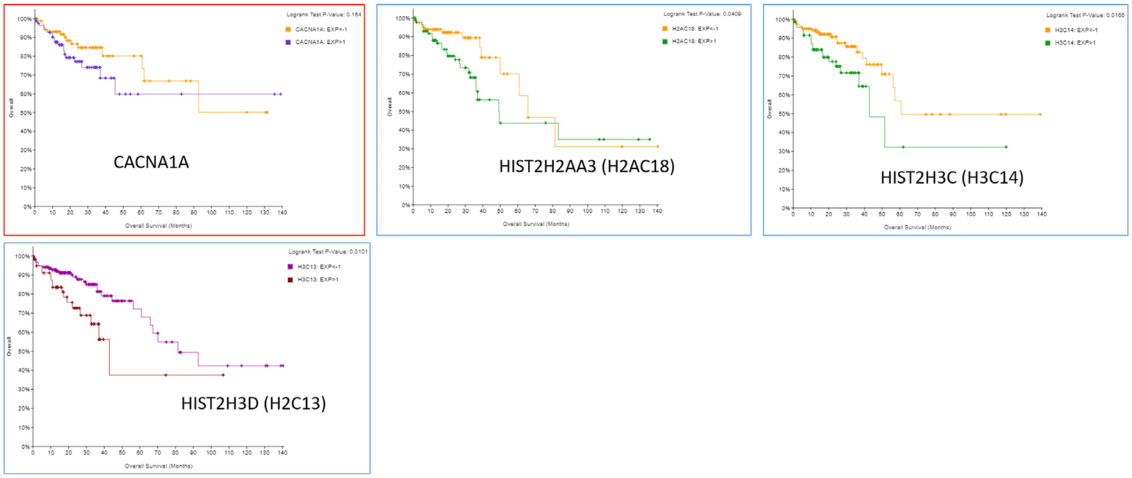

CPT-11-specific signatures

p53-independent

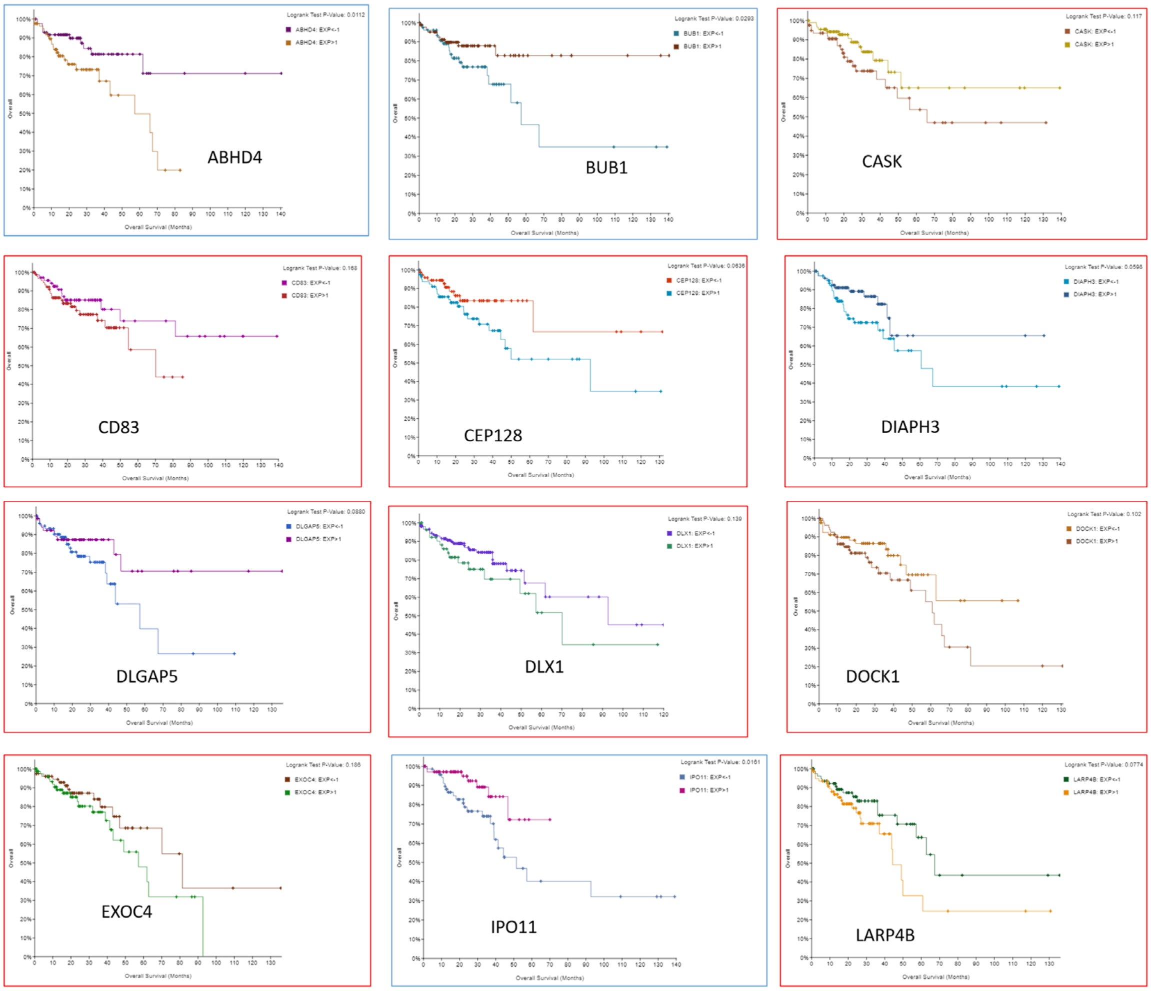

CPT-11-specific signatures

p53-independent

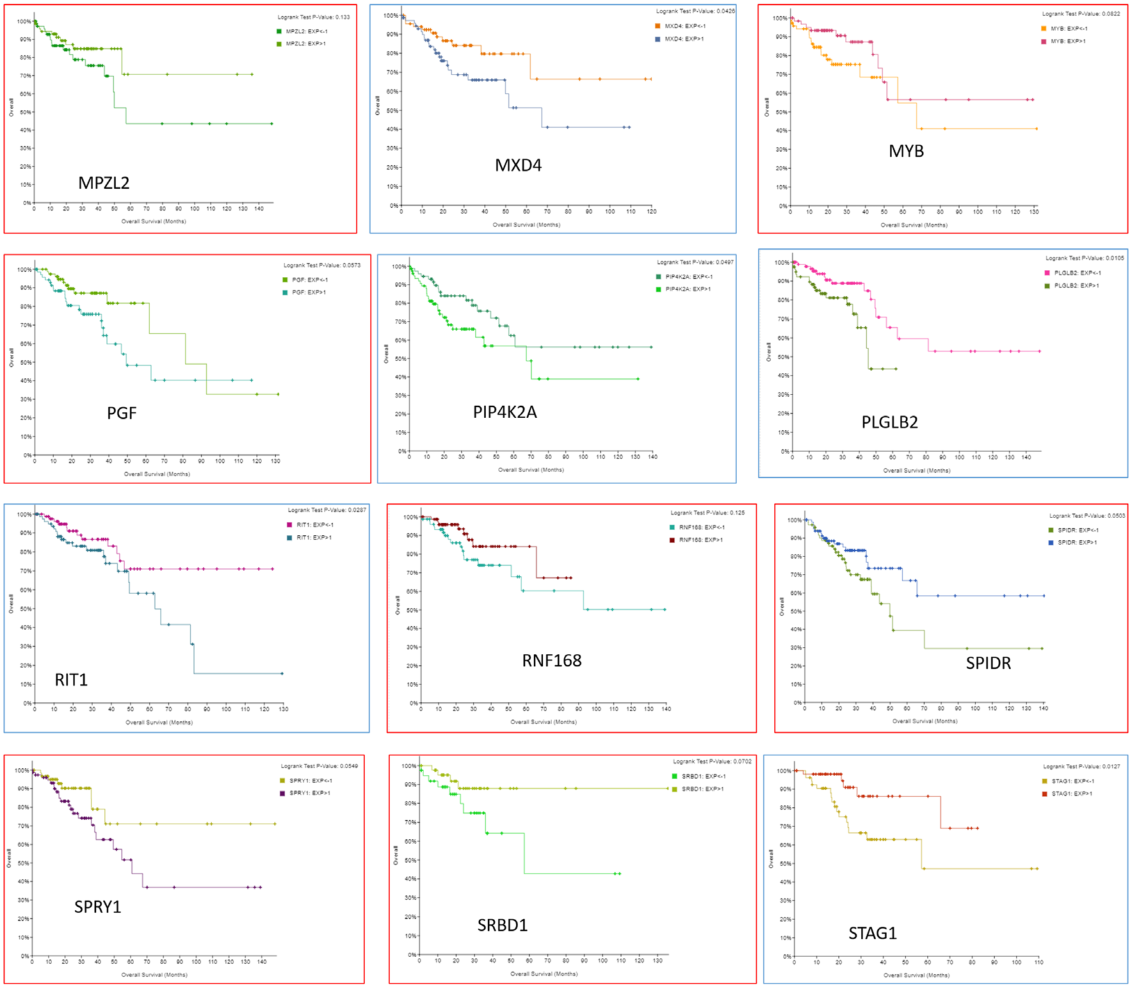

CPT-11-specific signatures

p53-independent

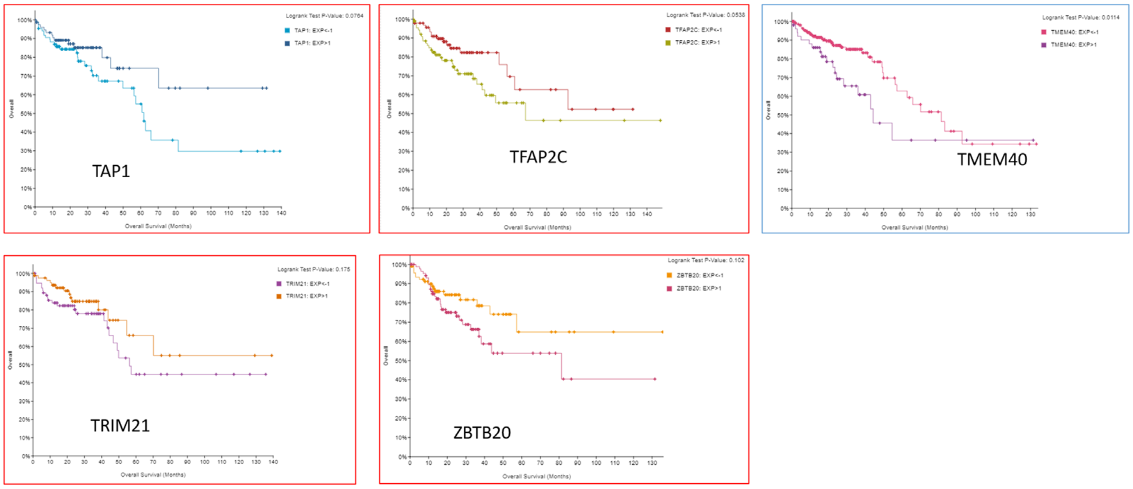

CPT-11-specific signatures

p53-dependent

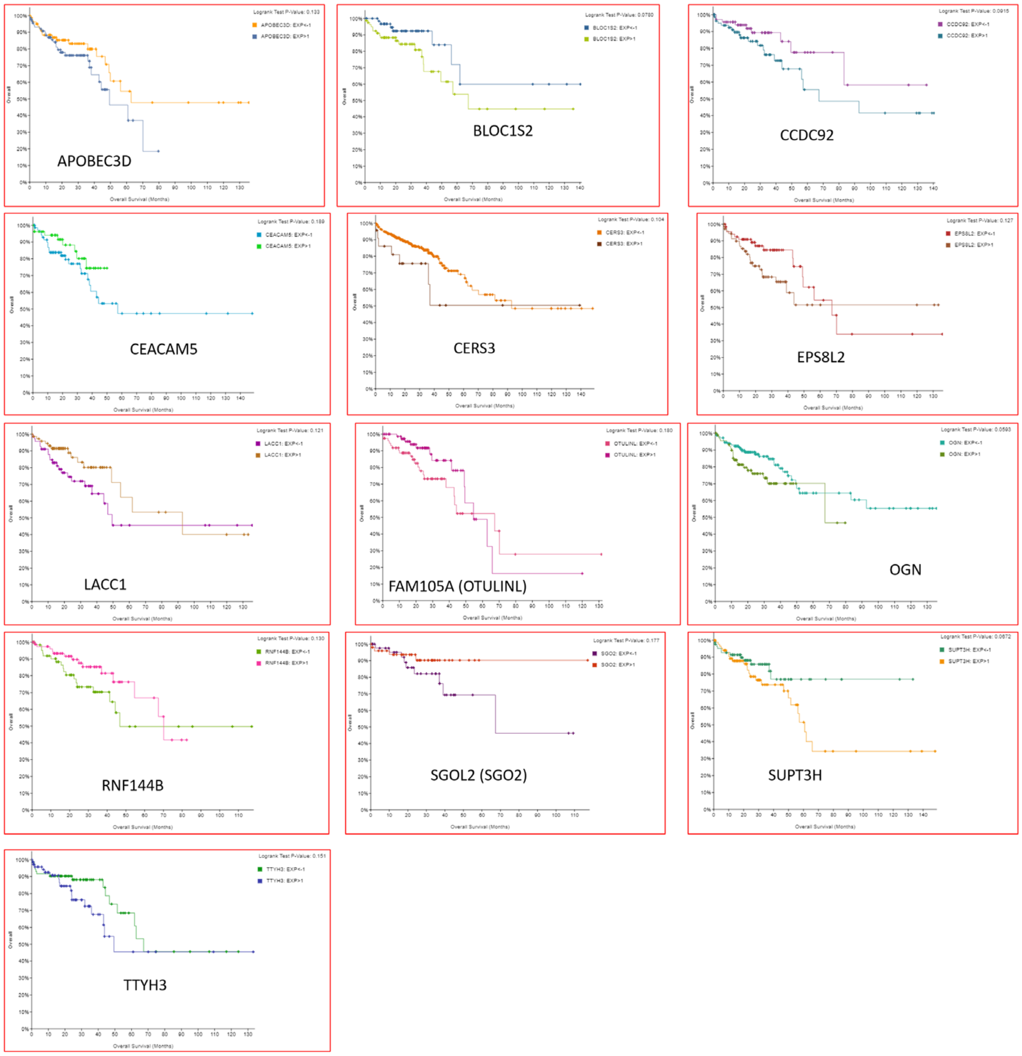

Oxaliplatin-specific p53-independent signature

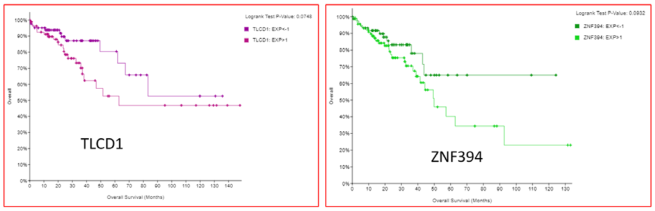

Cisplatin-specific p53-independent signature

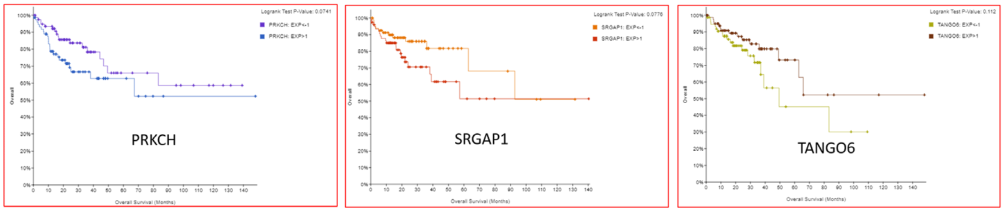

Supplementary Figure 5: Subsets of transcriptomic signatures in response to 5-FU, CPT-11, oxaliplatin, and cisplatin correlate with patient outcomes in colorectal cancer. TCGA (contains data on basal gene expression) was used to establish subsets of drug-specific signatures that correlated with patient outcomes. Difference in overall survival between patients with gene expression >1 or <−1 standard deviation from the mean was evaluated. Blue border = Logrank test *p*-value < 0.05. Red border = Logrank test *p*-value between 0.05–0.2.

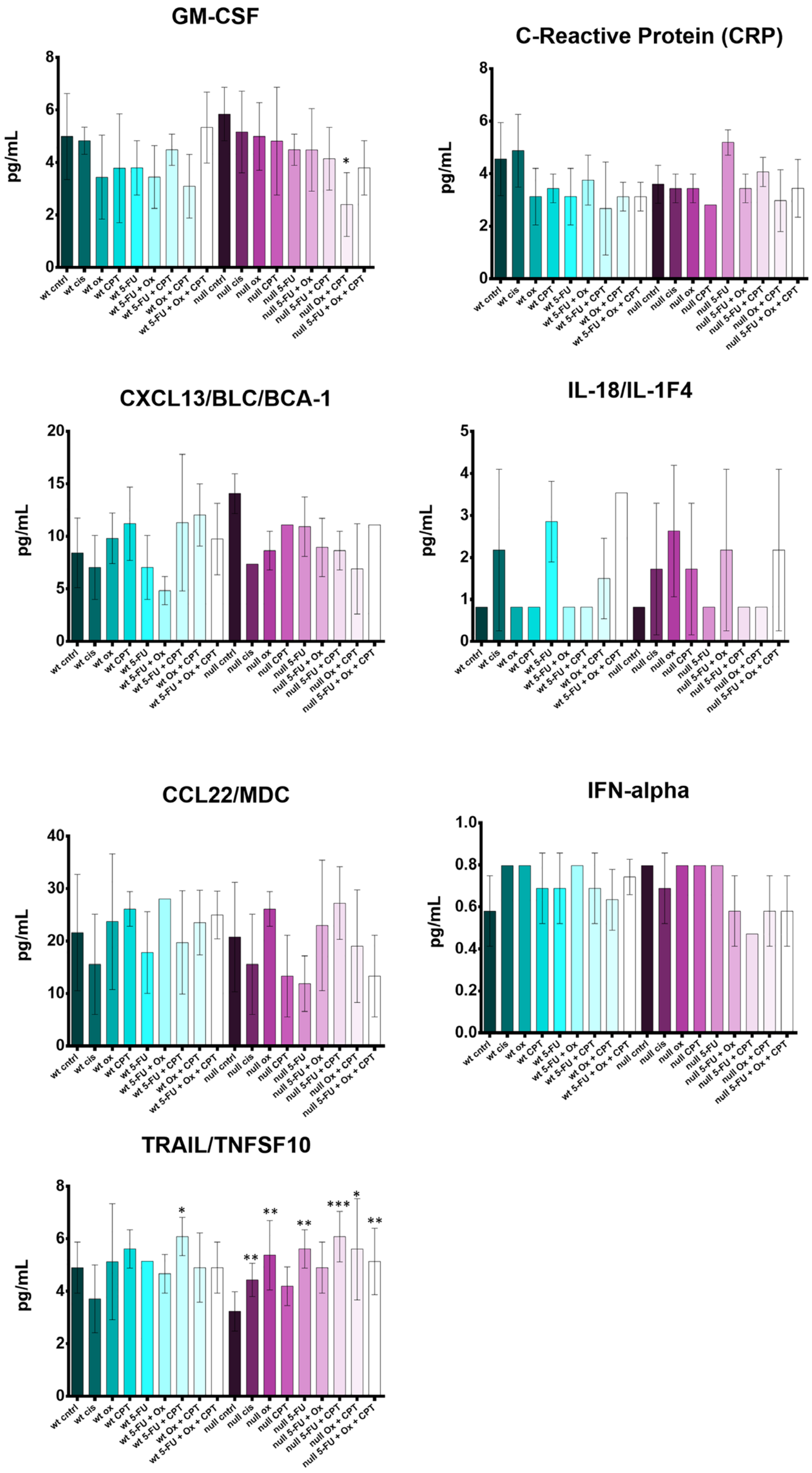

Supplementary Figure 6: Cytokine profiling reveals no significant effects on GM-CSF, CRP, CXCL13, IL-18, or CCL22. HCT116 and HCT116 p53−/− cells were treated at with the four drugs at their IC50s and combination treatment groups received 2–3 drugs, each at their individual IC50 concentration for 48 hours. Cytokine levels in the cell supernatants were measured with the Luminex 200 platform and significant differences (*p*-value <0.05 by one-way ANOVA) between control and treated groups was calculated.

Supplementary Table 1: Complete gene signatures and additional information for each gene in the master list (Probeset IDs, statistics, descriptions, etc.). See Supplementary Table 1
